# Unsupervised Deep Learning for Synthetic CT Generation from CBCT Images for Proton and Carbon Ion Therapy for Paediatric Patients

**DOI:** 10.3390/s24237460

**Published:** 2024-11-22

**Authors:** Matteo Pepa, Siavash Taleghani, Giulia Sellaro, Alfredo Mirandola, Francesca Colombo, Sabina Vennarini, Mario Ciocca, Chiara Paganelli, Ester Orlandi, Guido Baroni, Andrea Pella

**Affiliations:** 1 Bioengineering Unit, Clinical Department, CNAO National Centre for Oncological Hadrontherapy, 27100 Pavia, Italy; matteo.pepa@cnao.it (M.P.); giulia.sellaro@cnao.it (G.S.); 2 Department of Electronics, Information and Bioengineering, Politecnico di Milano (POLIMI), 20133 Milan, Italy; siavash.taleghani@mail.polimi.it (S.T.); chiara.paganelli@polimi.it (C.P.); guido.baroni@polimi.it (G.B.); 3 Medical Physics Unit, Clinical Department, CNAO National Centre for Oncological Hadrontherapy, 27100 Pavia, Italy; alfredo.mirandola@cnao.it (A.M.); mario.ciocca@cnao.it (M.C.); 4 Radiation Oncology Unit, Clinical Department, CNAO National Centre for Oncological Hadrontherapy, 27100 Pavia, Italy; francesca.colombo@cnao.it (F.C.); ester.orlandi@cnao.it (E.O.); 5 Paediatric Radiotherapy Unit, Fondazione IRCCS Istituto Nazionale dei Tumori (INT), 20133 Milan, Italy; sabina.vennarini@istitutotumori.mi.it; 6 Department of Clinical, Surgical, Diagnostic and Pediatric Sciences, University of Pavia, 27100 Pavia, Italy

**Keywords:** proton therapy, carbon ion therapy, adaptive particle therapy, paediatric oncology, CBCT, synthetic CT, artificial intelligence, deep learning, CycleGAN

## Abstract

Image-guided treatment adaptation is a game changer in oncological particle therapy (PT), especially for younger patients. The purpose of this study is to present a cycle generative adversarial network (CycleGAN)-based method for synthetic computed tomography (sCT) generation from cone beam CT (CBCT) towards adaptive PT (APT) of paediatric patients. Firstly, 44 CBCTs of 15 young pelvic patients were pre-processed to reduce ring artefacts and rigidly registered on same-day CT scans (i.e., verification CT scans, vCT scans) and then inputted to the CycleGAN network (employing either Res-Net and U-Net generators) to synthesise sCT. In particular, 36 and 8 volumes were used for training and testing, respectively. Image quality was evaluated qualitatively and quantitatively using the structural similarity index metric (SSIM) and the peak signal-to-noise ratio (PSNR) between registered CBCT (rCBCT) and vCT and between sCT and vCT to evaluate the improvements brought by CycleGAN. Despite limitations due to the sub-optimal input image quality and the small field of view (FOV), the quality of sCT was found to be overall satisfactory from a quantitative and qualitative perspective. Our findings indicate that CycleGAN is promising to produce sCT scans with acceptable CT-like image texture in paediatric settings, even when CBCT with narrow fields of view (FOV) are employed.

## 1. Introduction

Besides biological advantages, compared to conventional photon radiotherapy, particle therapy (PT) offers better conformity to the tumour target volume due to its sharp and steep dose gradients [1,2,3]. This feature makes particles the perfect candidate for paediatric patients with a long life expectancy, as they allow us to reduce long-term radiation-induced side effects. However, if on the one hand the enhanced conformal dose distribution resulting from the ballistic selectivity of particles is beneficial for the sparing of surrounding organs at risk (OARs), on the other hand it makes PT more susceptible to anatomical changes [4]. These are likely to occur as a combination of tumour shrinkage, weight loss, organ filling variation and displacement changes throughout the course of the treatment [5]. In particular, the pelvic and abdominal regions are highly prone to inter-fractional anatomical changes, due to the presence of organs with variable degrees of filling, including the gastrointestinal tract, bowel, rectum and bladder [6,7,8,9]. These variations can lead to reduced tumour coverage and/or healthy tissues overdosing, possibly undermining the oncological and toxicity outcomes of treatment [8,9].

To preserve the benefits of PT amid anatomical changes, the most common approaches include anatomical robust optimisation and plan adaptation [10]. The former considers non-rigid anatomical variations during the optimisation process, at the cost of an increased integral dose to healthy tissues [10]. Regarding the latter approach, to mitigate the possible detrimental effect of patient tissue changes on plan dosimetry, repeat computed tomography (CT) scans, also known as verification CT scans (vCT scans), are acquired during the course of treatment to recompute the plan on the current patient anatomy and trigger replanning if dosimetric objectives are no longer met [3,4]. In particular, it is estimated that about one third of PT patients will require at least one replanning during the course of their treatment [1]. However, the acquisition of vCT scans requires a strong clinical motivation, as they increase workload, in terms of room occupancy and staff time, and expose the patient to additional imaging doses [11,12]. Limiting the imaging dose is particularly important in sensitive patients such as paediatric patients with a good prognosis and life expectancy, to reduce additional complications to OARs as well as the risk of developing secondary malignancies [3,13].

Acquisition of vCT scans is not the only solution to obtain updated information on a current patient’s anatomy: the establishment of in-room, pre-treatment volumetric techniques for setup correction and image guidance has fostered the interest of the scientific community towards the possibility of their use for online dose calculation purposes. Magnetic resonance imaging (MRI) guidance has gained popularity in photon radiotherapy, while PT is at its infancy and limited to pre-clinical research settings, due to technical challenges related to the interaction of MRI with protons [11,14]. CT on rails is also promising, but increases the time and complexity of the treatment and constitutes an additional cost for the centre [15]. On the contrary, the availability of cone beam CT (CBCT) is definitely larger due to its lower cost and compactness [16], so it is routinely used worldwide for the daily verification and correction of the position of the patient. Since it is a volumetric imaging modality, it enables us to obtain valuable updated information daily on a patient’s anatomy quickly prior to radiation delivery and in treatment position [8].

Although CBCT imaging has the potential to adjust the treatment to the observed anatomical variations, the inherent lower image quality and contrast-to-noise ratio compared to CT and the consequent poor Hounsfield unit (HU) accuracy prevent its direct use for dose recalculation purposes in adaptive pathways [3,8,15,17,18,19]. In particular, the most relevant artefacts generally affecting CBCT images are beam hardening, aliasing and ring artefacts, a partial volume effect, as well as other ones arising from noise and scatter [20,21]. These can be prevented by means of pre-processing hardware solutions (e.g., bowtie filters, anti-scatter grids) or corrected via software post-processing methods either in the projection (i.e., on planar raw images, before reconstruction) or the image domain (e.g., on 3D volume, after reconstruction) [22,23]. The post-processing methods in the image domain are the most common to compensate for CBCT deficiencies and allow CBCT-based adaptive PT (APT) workflows [2], and can be roughly divided into two categories, namely analytical—also known as conventional—or data-driven, i.e., deep learning (DL)-based [22,24] methods. Amongst analytical methods, the most established technique to generate CT-like CBCT images, also denoted as *synthetic CT* (sCT), which is a deformable image registration (DIR) to planning CT (pCT) [8], alone [25,26] or in combination with other approaches, including HU mapping, histogram matching, multilevel threshold and empirical look-up table calibration [27,28].

Due to the recent unprecedented rise in artificial intelligence (AI) applications in healthcare and radiation oncology [29], DL-based methods have gained increasing popularity as a valuable tool for medical image synthesis, including CBCT to CT translation [8,11,22]. According to several authors, e.g., Dayarathana and colleagues, DL-based models can be broadly divided into supervised and unsupervised groups in terms of the learning method [30]. In computer vision, there is a large variety of models that are particularly utilised for image reconstruction and image synthesis [31]. Thanks to the recent development and advancements of generative adversarial networks (GANs) [32], it is feasible to synthesise images from input noise by simultaneously training two adversarial networks, a generator and a discriminator, as previously achieved by [33]. Another application of generative models that has attracted attention in recent years is image-to-image translation, that is generating synthetic images by translating an input image from a source domain to a target domain [34]. In this application, the model learns how to transfer style and underlying representations of the target domain to the content of the input image [34]. In the case of paired data availability, models such as pix2pix are employed to learn the mapping from one domain to the other in a supervised manner [35]. Unsupervised models, on the other hand, are advantageous when paired data (i.e., training data and ground truth) are not available [36]. In the context of medical image synthesis, several DL-based architectures for enhancing CBCT image quality were reported [37]. Among them, cycle consistency GAN (CycleGAN) has gained popularity as it allows us to use unpaired data for training, which is particularly important when CBCT and CT image pairs with completely matching anatomy are not available [17,23,24,38,39,40,41,42]. Despite the undisputable advances in the field, relevant open challenges still remain that prevent the routine use of DL and CycleGANs for image translation tasks in clinical settings and APT workflows [8].

In this work, we propose and evaluate a hybrid analytical and DL-based framework for CBCT to CT image synthesis tailored on a population of paediatric patients and young adults towards the implementation of APT pathways. In particular, this study focuses on an analytical ring artefact correction method followed by the generation of sCT scans based on 2D CycleGAN architectures featuring Res-Net and U-Net generators. The validity of the generated sCT scans is assessed in terms of qualitative and quantitative image quality, reserving dosimetric evaluations for future investigations. To the best of our knowledge, this study represents the first attempt to generate and evaluate DL-based sCT from narrow field-of-view (FOV) CBCT in a population of paediatric patients treated with either protons or carbon ions.

## 2. Materials and Methods

**Figure 1:**
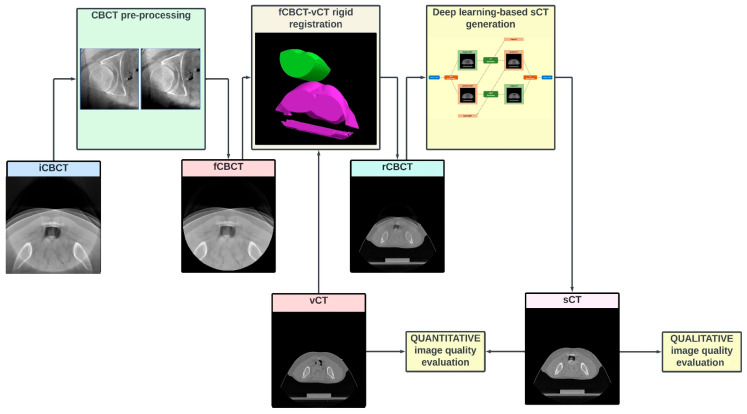
**Study flowchart.** Each CBCT volume (input CBCT, “iCBCT”) is pre-processed to (1) attenuate the ring artefact, (2) exclude the pixels outside the intrinsic CBCT circular field of view (FOV) and (3) intensity-match the CT grayscale. The pre-processed volume (filtered CBCT, fCBCT) is then resampled to match the geometrical CT scanning conditions and is rigidly registered (MATLAB intensity-based image registration) to same-day CT, i.e., verification CT (vCT), to obtain the CBCT registered on CT (rCBCT). After most cranial and caudal slices without CBCT are removed, and greyscale values are rescaled to a [–1, 1] range, the volume is inputted to the network to finally obtain the synthetic CT (sCT). Finally, image quality is assessed qualitatively and quantitatively.

The flowchart in Figure 1 describes the workflow of the study.

### 2.1. Patients Dataset

This retrospective study included a consecutive cohort of paediatric (age < 18 years old, yo) and young adult (18 ≤ age ≤ 30) patients treated between December 2021 and December 2023 at the National Centre for Oncological Hadrontherapy (CNAO) in Pavia, Italy. To be eligible for the study, the patients had to meet the following inclusion criteria: (1) age < 30 yo at time of treatment; (2) completion of a full PT course in CNAO; (3) treated for tumours within the pelvic or abdominal district, (4) imaged with in-room CBCT; (4) availability of clinical and imaging data (i.e., pCT and at least one vCT); (5) written informed consent for use of data for clinical research and educational purposes. Both treatments with protons and carbon ions were allowed. The study was approved by the local Ethical Committee “*Comitato Etico Territoriale Lombardia 6*” under notification number 0052020/23, prot. CNAO OSS 57 2023.

### 2.2. CBCT at CNAO

**Figure 2:**
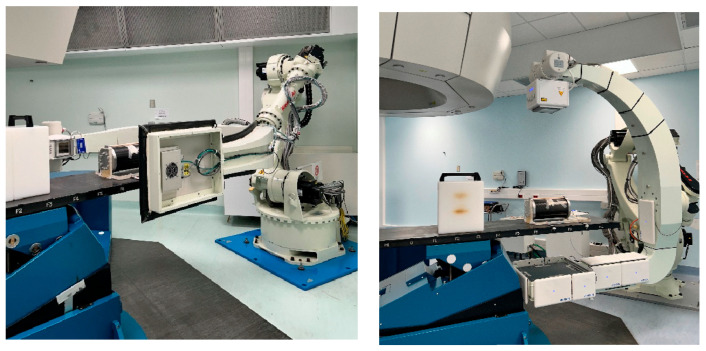
**Robotic CBCT devices in CNAO Rooms 2 (left) and 1 (right).** The former allows us to acquire exclusively full-fan (FF) (i.e., small FOV) CBCT scans, while the latter also enables half-fan (HF) (i.e., large FOV) acquisitions.

CNAO is a synchrotron-based facility able to deliver PT with fixed beam lines of protons and carbon ions species [43,44]. The Centre is equipped with three treatment rooms, two of them featuring custom, developed in house, robotic systems for the acquisition, in a *near-treatment* position, of planar X-ray projections for patient position verification based on bony landmarks and subsequent volumetric CBCT scans for qualitatively evaluating anatomical consistency with one of the pCT scans [45]. In one room (i.e., *Room 1*), it is possible to acquire either full-fan (FF) or half-fan (HF) CBCT [46], while in the other one (i.e., *Room 2*), only FF acquisition is enabled, limiting the field of view (FOV) to 220 mm (Figure 2). For the present study, only CBCT data from *Room 2* were considered, as the system in *Room 1* is in a pre-clinical phase. All data were entirely collected by our own research group at CNAO.

### 2.3. Imaging Data

The CT scans were acquired using a Siemens Somatom Sensation Open Bore scanner (Siemens, Germany), with an image size of 512 × 512 × (94–107) and voxel spacing of 0.9765 × 0.9765 × 2 mm^3^, with a grayscale range of [0, 4095]. The CBCT scans were acquired using a Varian A-277 X-ray tube and a Varian PaxScan 4030D flat-panel detector (Varian: Las Vegas, NV, USA) installed on the c-arm of the robotic imaging system [45]. About 600 projective images are acquired during a 220° gantry rotation around the patient and used to reconstruct the 3D volume by means of the Feldkamp–Davis–Kress (FDK) algorithm [45]. Image size was 220 × 220 × 220, voxel spacing was 1 × 1 × 1 mm^3^, and the greyscale range was [−1024, 1023]. For each patient, all available CT scans and CBCT scans acquired at a one-day distance at most were considered. If more than one CBCT scan was acquired in the same treatment session, only the most recent one was selected. This time constraint was deemed necessary to limit anatomical variability, thus enabling us to bypass DIR and perform a reliable and consistent CBCT-CT rigid registration (RR), as proposed by Rossi et al. on the same CBCT system [47]. As a matter of fact, DIR might introduce uncertainties and require proper validation, especially when the CBCT volume is flawed and has a narrow FOV [47].

### 2.4. Pre-Processing

**Figure 3:**
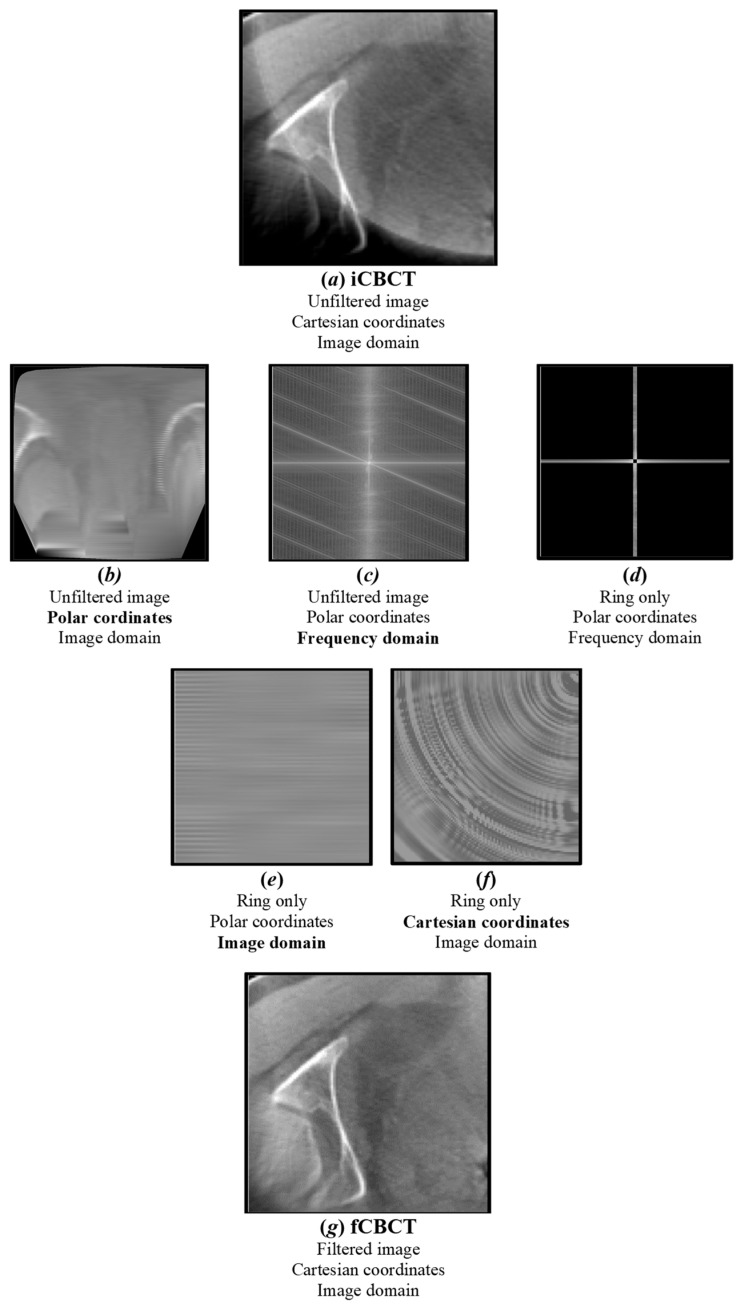
**Ring artefact reduction pipeline.** The original image (**a**) (iCBCT) is converted from Cartesian to polar coordinates (**b**) and its DFT is computed (**c**). A notch filter is applied to isolate the major ring’s frequencies (**d**), which are converted back to the image domain (**e**) and Cartesian coordinates (**f**). The output filtered image (**g**) (fCBCT) is obtained by subtracting the ring from the original image.

All images were pre-processed using MATLAB R2023b (The MathWorks Inc., Natick, MA, USA) before being presented to the DL network. First of all, all CBCT images were pre-aligned to the CT coordinate system to obtain input CBCT (iCBCT). After this, a ring artefact attenuation method was applied by transforming the images from Cartesian to polar coordinates and by isolating the characteristic signature of the ring in the frequency domain by means of discrete Fourier transform (DFT) (Figure 3), following these steps:

1. Each CBCT slice is converted from Cartesian to polar coordinates using interpolation [48]. In Cartesian coordinates, the ring manifests itself as a series of concentric rings, with the centre coinciding with the reconstructed image centre. In polar coordinates, instead, it is converted into parallel horizontal stripes [49].
2. The DFT of the CBCT in polar coordinates is computed: horizontal stripes appear as a vertical band in the middle of the whole spectrum, i.e., their high-frequency information is located at the centre position of the image [49].
3. Low-pass filtering in the frequency domain is used to remove artefacts while the high-frequency details of the image are preserved [49].
4. The ring’s frequencies are converted back from frequency to imaging domain-polar coordinates, appearing as a series of parallel stripes. An anisotropic Gaussian filter is also applied to smooth stripes in the horizontal direction only.
5. The ring in polar coordinates is converted back to Cartesian coordinates, clamping its values in the [−10 10] range, in order not to significantly affect the original CBCT grey-level distribution.
6. The ring in Cartesian coordinates is algebraically subtracted to the original image to obtain a new image with the ring artefact significantly reduced.

At the end of this pipeline, a filtered CBCT (fCBCT) is obtained. Before CBCT-CT registration, the CBCT grayscale was shifted to approximate that of the CT, that is, [0, 4095]. This was achieved by shifting all CBCT values in the positive direction by their minimum values; in this way, minima of CBCT and CT coincide and are equal to 0. Even if CycleGAN does not require one-to-one registered voxel information, before feeding the data to the network, a resampling of CBCT imaging data and a rigid registration of CBCT on CT was performed, as proposed in [24], to obtain the registered CBCT (rCBCT) volume. The voxel spacing and image size of pre-processed data were consequently unified as those of CT, that is, 0.9765 × 0.9765 × 2 mm^3^ and 512 × 512 × (94–107), respectively. As the scanner range of CT was, in general, longer than that of CBCT, to balance the anatomic extent in the longitudinal direction, CT slices beyond the CBCT FOV were removed, as seen in [17]. Additionally, as the two most cranial and caudal CBCT slices were often truncated, they were removed systematically for all volumes, as also proposed by [8]. For the sake of computational efficiency and faster convergence, all images were then normalised to a (−1, 1) range, as suggested by [50]. This kind of normalisation preserves the original image contrast [50].

### 2.5. CycleGAN

A CT synthesis problem under a DL framework was formulated in this study. CycleGAN has been selected as it allows us to perform an image-to-image translation task using unpaired data [8,23,24,38,39,40,42]. This choice was mandatory in this dataset as a proper ground truth was not available, as CBCT scans and CT scans, even if acquired one day apart at most, still presented a certain degree of anatomical variation.

**Figure 4:**
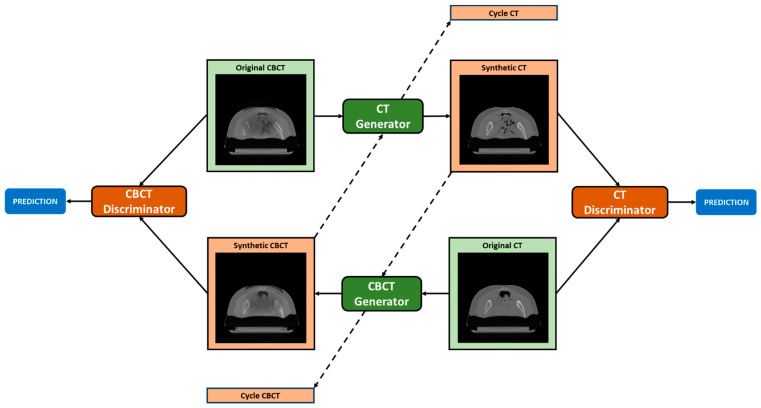
**CycleGAN topology.** The CT generator converts original CBCT into pseudo-CT (*Synthetic CT*) and pseudo-CBCT back to CT (*Cycle CT*). The CBCT generator converts original CT into pseudo-CBCT (*Synthetic CBCT*) and pseudo-CT back to CBCT (*Cycle CBCT*). CBCT and CT discriminators are employed to judge the authenticity of generated pseudo-CBCT and pseudo-CT images. The loss obtained from the discriminators is used to update the weights of both the generator and discriminator networks.

The particular design of CycleGAN, which consists of two generators and two discriminators, enables bidirectional translation between CBCT and CT domains. Each generator is trained to generate images of a specific domain, while fooling the discriminator of the corresponding domain that is responsible for distinguishing fake (i.e., synthetic) and real images in an adversarial manner. The assumption is that the generator, by learning the mapping from the source to the target domain, is capable of generating images that are similar to target domain images and have an ideally identical distribution as of the empirical distribution of the target domain data. In particular, the CT generator is responsible for generating pseudo-CT images (i.e., sCT) from CBCT images, and the CT discriminator is employed to distinguish the sCT scans from real CT scans (i.e., vCT scans), in a binary fashion, i.e., 1 = real, 0 = fake. The consistency of image translation from one domain to the other one is established by the cycle consistency concept proposed by [51]. Cycle consistency implies that if one input image is translated to the other domain and then translated back to the original domain, the output must be ideally identical to the initial input. For instance, one original CBCT is given to the CT generator and then the sCT (which is the output of the CT generator) is translated back to the CBCT domain through the CBCT generator; it prevents the two learned mappings (CBCT to CT and vice versa) from contradicting each other [51]. In order to maintain that the HU value of the generated image is as similar as possible to the target domain, identity mapping is also incorporated. Accordingly, the CT generator takes as input CT images, which are assumed to remain unchanged after going through the generator. The overall network structure is depicted in Figure 4.

To achieve the above-mentioned goals (matching distributions, contradiction prevention and HU preservation), the following losses are presented in the objective. The two first terms ($L_{GAN-G}\;and\;L_{GAN-F}$, Equations (1) and (2)) are adversarial losses that are applied to mapping functions from CBCT to CT and vice versa, respectively. This is a min-max competition in the sense that the generator tries to minimise this term by generating images that are as similar as possible to those of the target domain, while the discriminator aims to maximise the term by distinguishing fake and real images. Additionally, it employs cycle consistency loss ($L_{CC}\left(G,\;F\right)$, Equation (3)) to ensure mappings are bijective, allowing for the reconstruction of original images from translated versions. Finally, identity loss ($L_{identity}\left(G,\;F\right)$, Equation (4)) is added to emphasise HU preservation between vCT and sCT, and between CBCT and synthetic CBCT. The full objective (*L*, Equation (5)) combines the adversarial losses, the cycle consistency loss and the identity loss, and parameters α and β control the relative importance. In the equations, G is the CT generator, F is the CBCT generator, D_CT_ is the CT discriminator, D_CBCT_ is the CBCT discriminator, and E is the expected value.

(1) $$L_{GAN-G}\left(G,\;D_{CT},CBCT,\;CT\right)=E_{CT\sim Pdata(CT)}\left[\log D_{CT}\left(CT\right)\right]+E_{CBCT\sim Pdata(CBCT)}\left[\log(1-D_{CT}\left(G(CBCT)\right)\right]$$

(2) $$L_{GAN-F}\left(F,\;D_{CBCT},CT,\;CBCT\right)=E_{CBCT\sim Pdata(CBCT)}\left[\log D_{CBCT}\left(CBCT\right)\right]+E_{CT\sim Pdata(CT)}\left[\log(1-D_{CBCT}\left(F(CT)\right)\right]$$

(3) $$L_{CC}\left(G,\;F\right)=E_{CBCT\sim Pdata(CBCT)}\left[{∥F(G(CBCT))-CBCT∥}_{1}\right]+E_{CT\sim Pdata(CT)}[{∥G(F(CT))-CT∥}_{1}]$$

(4) $$L_{identity}\left(G,\;F\right)=E_{CT\sim Pdata(CT)}\left[{∥G(CT)-CT∥}_{1}\right]+E_{CBCT\sim Pdata(CBCT)}[{∥F(CBCT)-CBCT∥}_{1}]$$

(5) $$L\left(G,F,D_{CT},D_{CBCT}\right)=L_{GAN-G}\left(G,\;D_{CT},CBCT,CT\right)+L_{GAN-F}\left(F,\;D_{CBCT},CT,CBCT\right)+\alpha L_{CC}\left(G,\;F\right)+\beta L_{identity}\left(G,\;F\right)\;$$

**Figure 5:**
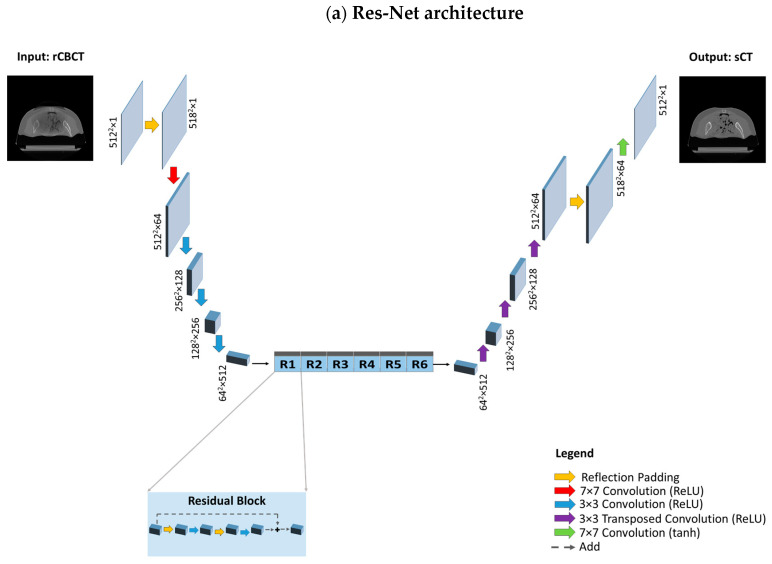

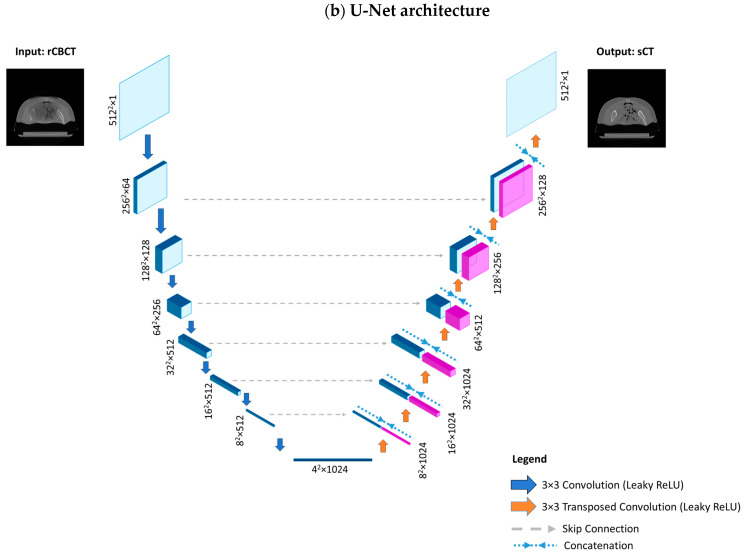
**Model architectures.** (**a**) Res-Net architecture, composed of a downsampling path, a stack of residual blocks and an upsampling path. The downsampling path reduces the spatial dimension from 512^2^ to 64^2^ through convolution layers, while increasing the depth from 1 to 512. Each residual block, consisting of reflection padding and convolution layers, provides as output the sum between the input activation map and the output of the last convolution layer. The upsampling path is composed of transposed convolution layers which increase the spatial dimension to restore the initial size (from 64^2^ to 512^2^), while reducing the depth from 512 to 1. (**b**) The U-Net architecture is composed of a downsampling and an upsampling path; however, instead of residual blocks, there exist skip connections that directly transfer information through concatenating the activation maps from the donsampling path to the corresponding activation map with the same spatial dimension and depth in the upsampling path.

Two different architectures that are commonly utilised for image reconstruction were investigated for generators, namely Res-Net and U-net, as previously performed by [8,52]. In the former, the encoding part is composed of 2D convolution layers (kernel size 3 × 3, strides 2) followed by instance normalisation and ReLU as the activation function. It features symmetric downsampling and upsampling layers holding 6 residual blocks in between; each residual block is composed of 2D convolutional blocks (kernel size 3 × 3, strides 1), instance normalisation and reflection padding (1, 1). The decoder part of the network consists of 2D transpose convolution layers to restore the input shape of 512 × 512 × 1, zero padding, instance normalisation, and ReLU as the activation function. Concerning the last layer, convolution (kernel size 7 × 7, strides 1) is applied. Regarding U-net, skip connections concatenated from corresponding encoding layers help to preserve the spatial information lost in the contracting path, which facilitates locating features more accurately within the decoding path. Both discriminators have a similar structure and are composed of 2D convolutional blocks (kernel size 4 × 4, stride 2), zero padding, instance normalisation, and Leaky ReLU. Res-Net and U-Net architectures are depicted in Figure 5.

Regarding training configuration, the model was implemented in a dedicated workstation using Python 3.10.11, TensorFlow 2.10.0 and NVIDIA RTX A4000 graphical processing unit (GPU) (NVIDIA Santa Clara, CA, USA). A batch size of 2 was tried for U-Net and 1 for Res-Net. The training time per epoch was slightly over 25 min for U-Net and 105 min for Res-Net. An Adam optimizer was applied for optimisation and a random normal initializer was recruited to initialise network weights. The model was trained on 2D images of a size of 512 × 512 × 1.

### 2.6. Evaluation Metrics

In the context of APT, the quality of generated sCT images is commonly assessed either using image or dose similarity metrics [37]. In this work, the goodness of the generated sCT images is evaluated using image metrics only, as dosimetric evaluations will be the object of future investigations.

Among image similarity metrics, structural similarity (SSIM) and the peak signal-to-noise ratio (PSNR) were selected, being sufficiently robust towards anatomical variations between a reference image (i.e., vCT) and an examined image (i.e., sCT), which are likely to occur in the dataset herein considered.

SSIM is able to quantify the similarity between a couple of images as a combination of luminance (l), contrast (c) and structure (s) [53,54]:

(6) $$SSIM\left(x,y\right)={\left[l\left(x,y\right)\right]}^{\alpha }\cdot {\left[c\left(x,y\right)\right]}^{\beta }\cdot {\left[s\left(x,y\right)\right]}^{\gamma }$$

where

(7) $$I(x,y)=\frac{2\mu_{x}\mu_{y}+C_{1}}{\mu_{x}^{2}+\mu_{y}^{2}+C_{1}}$$

(8) $$c(x,y)=\frac{2\sigma_{x}\sigma_{y}+C_{2}}{\sigma_{x}^{2}+\sigma_{y}^{2}+C_{2}}$$

(9) $$s(x,y)=\frac{2\sigma_{xy}+C_{3}}{\sigma_{x}\sigma_{y}+C_{3}}$$

Here, α, β and γ are positive constants, μ_x_ and μ_y_ are the local means, σ_x_ and σ_y_ are the standard deviations and σ_xy_ is the cross-covariance for images x and y. SSIM varies between 0 and 1, with higher values corresponding to higher image similarity [24].

The PSNR is computed as the ratio between the maximum signal power (P_max_) and the noise affecting the quality of representation (root mean square error, RMSE), and is expressed in decibels (dB) [37].

(10) $$PSNR=20\;{log}_{10}\frac{P_{max}}{RMSE}\;[dB]$$

The sCT image quality was also assessed qualitatively by the visual inspection of the output image. The following image characteristics were investigated: image texture and contrast, the fidelity to the original anatomy and the presence of artefacts.

## 3. Results

### 3.1. Patients’ Data

Out of the 501 screened patients (<30 yo as of 31st December 2023), 21 (12 paediatric patients and 9 young adults, median age 17 yo) met the inclusion criteria and were included in the study. Nine were treated with protons, the remainder with carbon ions, with different treatment schedules. For all of them, pCT scans and vCT scans were exported in the DICOM format and were anonymized from the RayStation 11B (RaySearch Laboratories AB, Stockholm, Sweden) treatment planning system (TPS), while CBCT volumes and relative metadata were collected and anonymized from the dedicated hospital repository.

### 3.2. Training–Testing Split

Of the 55 same-day CBCT scans collected from the 21 patients, 44 (15 patients) were included in the study, and were divided into training (36 volumes, 3820 slices, 10 patients) and testing (8 volumes, 812 slices, 5 patients) sets. As a general rule, patients with more eligible CBCT scans available were included in the training set, to provide more data to the model to better learn the diverse features and representations while still guaranteeing a sufficient number of unseen test cases from the rest of the patients. Only 5 of the 44 volumes were produced with CT scans made at a one-day distance with respect to the CBCT; the remainder were acquired exactly on the same day. Eleven volumes (6 patients) were discarded due either to CT import issues, registration failure, or major image quality problems (Table 1 and Figure S1).

### 3.3. Pre-Processing Outcomes

**Figure 6:**
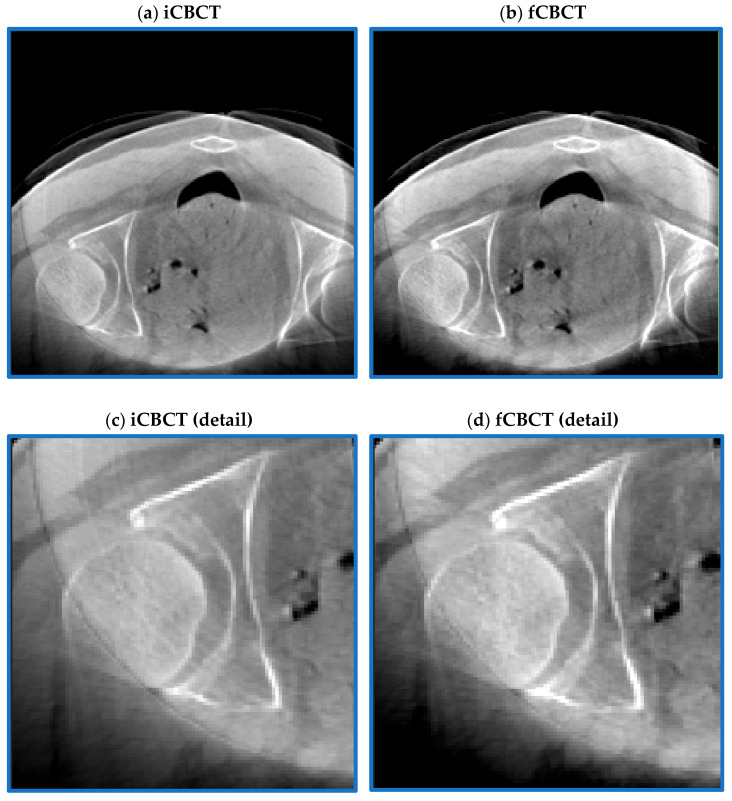
**Pre-processing outcome.** CBCT before (**a**) and after (**b**) the ring artefact reduction procedure. The magnification shown in the two bottom figures further highlights how the stripes typical of the ring artefact (**c**) are strongly reduced in (**d**) while maintaining an acceptable image contrast.

Despite some residuals in the centre of the image, the ring artefact was strongly reduced while contrast and details remained almost unaltered (Figure 6). Registration was acceptable except for three cases, which were excluded from the analysis.

### 3.4. Qualitative Evaluation

**Figure 7:**
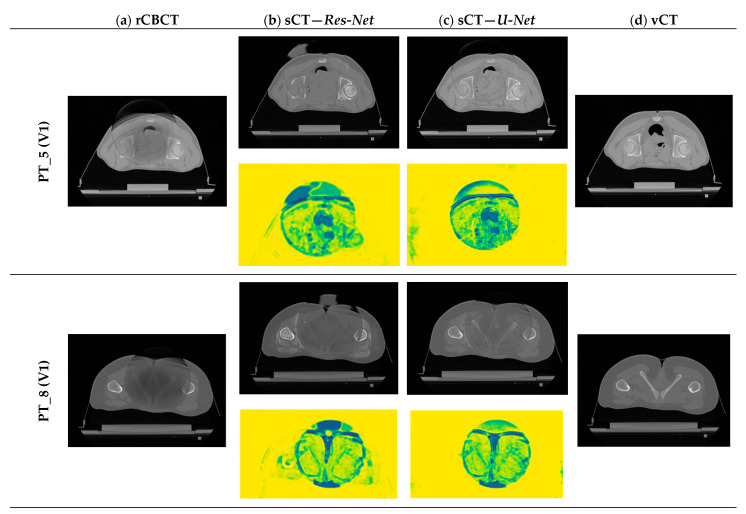

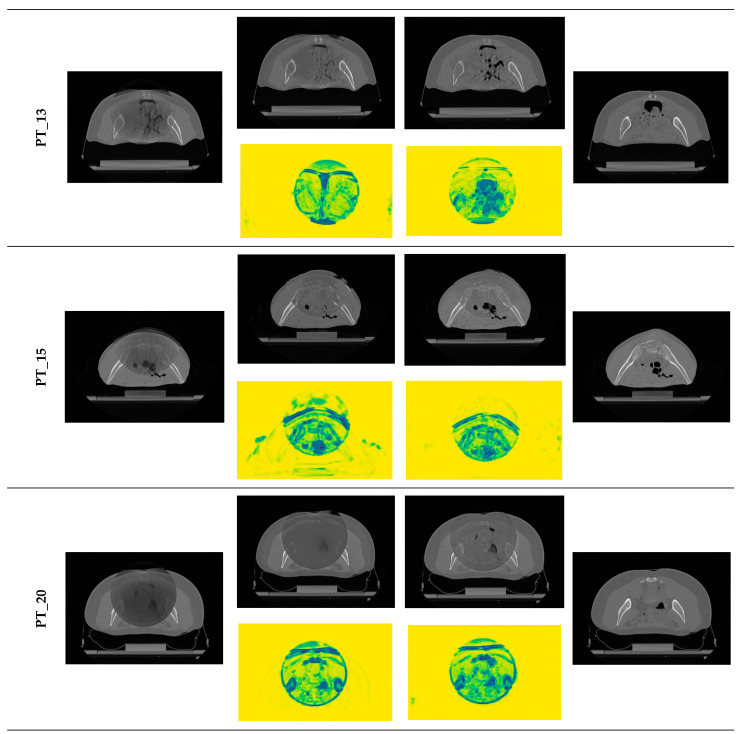
**Examples of generated sCT scans.** Examples of one representative slice for one sCT volume of all testing patients, generated from rCBCT (column a) using both Res-Net (column b) and U-Net (column c). Similarity maps (yellow background) are also shown: the degree of modification is given by the colour scale: yellow = 1 (i.e., no change, highest similarity), dark blue = 0 (i.e., highest discrepancy).

Qualitative evaluation is of paramount importance in exploratory studies assessing the performance of a network performing image-to-image translation tasks, in order to detect at a glance evident major issues beyond numerical results, as shown in [55]. To visually evaluate the performance of the proposed sCT generation infrastructure, some representative sCT slices and similarity maps of all testing patients (one volume per patient) are shown in Figure 7, for both Res-Net and U-Net.

**Figure 8:**
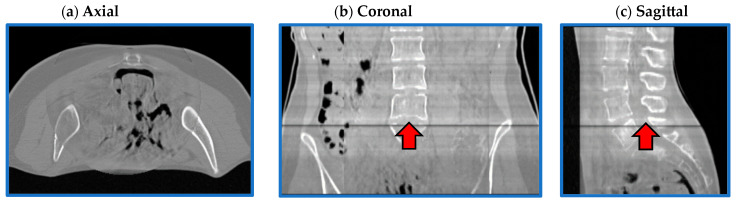
**Flaws in coronal and sagittal views.** Axial (**a**), coronal (**b**) and sagittal (**c**) views from one representative sCT (PT_13). Image intensity appears uniform in the axial plane, while it slightly varies in the coronal and sagittal views. In (**b**,**c**), it is evident that the intensity of one slice was significantly different to the adjacent ones.

For both architectures, although CBCT and CT pairs were acquired on the same day or at most one day apart, some degree of anatomical variation was observed for all patients. Regarding image quality, it is evident that the sCT image texture approximates better that of vCT if compared to the original CBCT. In particular, soft tissues’ contrast is enhanced, air pockets are crisper and bones are sharper. High-frequency artefacts, such as streaks, were not removed completely but were significantly suppressed. CBCT FOV edges appear attenuated but remain visible. As hypothesized, the similarity maps, depicting the residual image between input CBCT on CT and sCT, witness that most changes (dark dots) brought by the network occur within the CBCT FOV and involve air and bones. It also emerges that the quality of the sCT depends on the quality of the input CBCT or pre-processing. In the examples in Figure 7, it can be appreciated that for patients 5 and 13, the better rCBCT appearance reflects on the better sCT image quality. Slight intensity variations between adjacent slices occur, as a consequence of the 2D implementation of the network, that synthesise the sCT not as a 3D volume but as a series of consecutive 2D slices (Figure 8).

Finally, when comparing Res-Net with U-Net, it is evident that the latter provides a better delineation of the air–tissue interface as well as better soft tissue contrast in this setting of patients.

### 3.5. Quantitative Evaluation

The SSIM and PSNR values used to quantify image quality for both Res-Net and U-Net are reported in Table 2. Results are reported as the median (across slices) and interquartile range for all patients and volumes. A visual representation is also provided in Figures S2 and S3. SSIM values were computed for all rCBCT-vCT and sCT-vCT pairs, to assess the degree of improvement brought by the CycleGAN. The analysis focused exclusively on the area enclosed by the original CBCT, in order to exclude CT values outside, that are expected to be unaffected by the network. A Wilcoxon signed-rank test was performed to assess if any possible difference between input and output images in terms of the SSIM and PSNR was statistically significant (*p*-value < 0.05). This non-parametric test was employed as it does not require a normal distribution of the two populations. The statistically significant different distributions of SSIM and PSNR before and after CycleGAN processing, for all patients but one, witness the improvements brought on by the network. For patient 8, no statistically significant differences were observed between U-Net SSIM values.

## 4. Discussion

In this work, we have proposed and evaluated a CycleGAN-based approach to translate daily acquired CBCT scans into sCT scans in a cohort of paediatric patients and young adults treated with proton or carbon ion therapy. Even if the clinical validity for dose calculation purposes has not been proven yet, the satisfactory results in terms of qualitative and quantitative image quality suggest the potential usefulness of these sCT scans in APT pathways.

In particular, two generator architectures have been investigated, with one of them (i.e., U-Net) outperforming the other one (i.e., Res-Net) in the considered dataset, with the described pre-processing and with the chosen hyper-parameters’ configuration. Overall, sCT scans obtained from U-Net contain more details and, from an anatomical point of view, images are more realistic. In addition, this network almost does not change the regions in the input image that correspond to real CT values, indicating that it succeeds in fulfilling the identity mapping objective. On the other hand, Res-Net shows some deficiencies in effectively reproducing the air–body interface, soft tissues and bony anatomy. It was also evident from results that the quality of the generated sCT is highly dependent on the quality of the input images and pre-processing. Specifically, stronger artefacts affecting the image as well as poorer tissues contrast during the acquisition phase, which, although attenuated, still remained visible. Nevertheless, no evident flaws like the ones reported in [55] were observed.

This work presents several strengths that are worth acknowledging. First of all, few studies employ CBCT scans with a FOV smaller than the patient’s body in the axial plane [56]. Additionally, Rossi et al. applied deep learning algorithms to narrow-FOV truncated CBCT scans acquired with the same system but exclusively for image quality improvement [47]. Furthermore, in that study, CT volume was cropped to CBCT FOV, while here, CBCT volume was embedded in CT. Additionally, the framework was tailored for a population of paediatric patients with pelvic malignancies, which is a challenging application due to the high inter-fraction variability due to bowel filling conditions [8]. Indeed, this is one of the few studies on CBCT correction that includes paediatric data [3,8,17]. As a matter of fact, it is more common to use MRI to CT conversion for paediatric patients [3]. However, one comparative study on an adult head and neck cancer population demonstrated the non-inferiority of CBCT-based sCT to the MRI-based counterparts for dose calculation purposes for APT applications [11]. The choice of the paediatric population was mainly driven by two factors: the first was related to the clinical significance of the study, as younger patients are more sensitive to the imaging radiation dose and CBCT-based sCT could in theory avoid unnecessary vCT, and the second factor was related to technical reasons, as a smaller patient’s body is more suitable for narrow FOVs. The availability of a CBCT system fully developed in-house brings extraordinary flexibility and potential groundbreaking advantages as it allows us, in principle, to (1) explore new strategies based on raw data and (2) to easily streamline the sCT generation and APT process in future implementations. Another peculiarity characterising this study is training the model on spurious CBCT images, i.e., CBCT scans embedded in CT scans. Admittedly, most studies in the literature use pure CBCT scans as input for the networks [41,42,57,58]. However, the same studies employ large FOV CBCT images, that easily include the whole body part considered. Furthermore, other investigations exist that incorporate CT information as input [50]. In our case, this choice was driven by the final clinical endpoint of the study, i.e., producing CT-like images to be used for evaluating anatomical changes and for dose recalculation purposes. Our results demonstrated that this approach was successful from several points of view. As a matter of fact, we observed that changes occurred mainly within the CBCT FOV, demonstrating that the model effectively learned the mapping from the CBCT domain to the CT domain. To conclude, it is worth mentioning that, even though training was performed on CBCT images registered with and embedded in CT scans, similarity metrics were evaluated exclusively within the CBCT FOV: in this way, it was possible to establish the actual performance of the networks in mapping CBCT images into the CT domain.

Despite being innovative in nature, this study presents some limitations which are worth acknowledging. First of all, the sample size is small in consideration of the huge amount of data that is typically required for training deep convolutional neural networks (DCNNs). However, the number of paediatric patients treated for abdominal or pelvic tumours is known to be small in radiation oncology departments [17] and is a common issue in all similar studies involving this setting of patients (ours: 15 patients, 44 volumes; Sheikh et al. 2022 [3], Ates et al. 2023 [59]: 10 patients/10 volumes; Uh et al. 2021 [17]: 64 patients/64 volumes; Szmul et al. 2023 [8]: 64 patients/209 volumes, but in conventional radiotherapy). Another important limitation is the lack of a proper ground truth, which prevents us from performing a voxel-wise evaluation of image quality also in terms of HU accuracy, by means of metrics such as mean absolute error (MAE) or mean square error (MSE). Qiu et al. performed DIR between pCT and CBCT to produce perfectly paired data and employ supervised approaches [22]. However, as correctly pointed out by Szmul et al., achieving accurate DIR results is challenging, especially in the pelvic district characterised by important bowel gas changes [8]. In addition, from a DL perspective, the unavailability of paired data is not an issue, if unsupervised approaches, like the one herein investigated, are employed [8]. To make up for the absence of a gold standard, future implementations of the project could foresee the use of computational phantoms to ensure the perfect correspondence between rCBCT and vCT, as also suggested by Parrella et al. for MRI applications [60].

Another minor limitation is the occasional intensity variations between consecutive slices. This issue was not unexpected, as a 2D architecture was employed with no specific constraints enforced between adjacent slices, and can be easily solved using appropriate post-processing methods. Similar issues in the cranio-caudal direction were reported in similar studies in the literature employing 2D networks [8,24]. Overall, no major defects in axial and coronal planes were observed, as reported instead in [24].

Overall, the proposed sCT generation pipeline can be further improved by optimising pre-processing and fine-tuning the network’s hyperparameters. This can be particularly true for the Res-Net architecture, which significantly underperformed compared to the other one. Our choice was to pre-select the best pre-processing parameters and the network’s hyperparameters so that results from different architectures and configurations were more easily comparable [8].

Regarding PT_08, the decrease in performance can be ascribed to the different anatomical district considered for this patient (lower limbs) compared to that of the patients used to train the model (pelvis). This is a perfect example of how the low sample size has negatively impacted on the generalizability capabilities of the model.

SSIM and PSNR values before (rCBCT vs. vCT) and after (sCT vs. vCT) witness the image quality boost brought on by the network for most patients. From a quantitative standpoint, excluding PT_08, the average median SSIM increased from 0.72 (rCBCT vs. vCT) to 0.78 and 0.75 (sCT vs. vCT) for Res-Net and U-Net, respectively. Similarly, the average median PSNR value augmented from 47.77 (rCBCT vs. vCT) to 51.51 and 50.21 for Res-Net and U-Net, respectively. Our results are comparable with the published literature. Chen and colleagues [50] reported SSIM and PSNR values increasing from 0.9079 to 0.9642 and from 26.74 to 30.75, respectively, after feeding the images to a U-net-based model. Similarly, Liang and colleagues [41] reported SSIM and PSNR values increasing from 0.73 to 0.85 and from 25.28 to 30.65, respectively, employing CycleGAN to generate sCT from CBCT. As mentioned earlier, it was not possible to assess the absolute quality of sCT due to the absence of a true gold standard.

This study represents a preliminary investigation that paves the way to a large variety of possible future directions, including the following:

- Training and testing sets’ enlargement by including adult patients and employing data augmentation techniques—it is expected that by increasing the sample size, the performance of the network will improve;
- Validation on other anatomical areas, as previous investigations have demonstrated the inter-anatomic generalizability of DCNNs [17];
- Validation on pelvic and extra-pelvic images acquired in both FF and HF modalities using the newly developed system in CNAO Room 1, as soon as first data are available;
- Validation on publicly available data repositories to assess the performance of the network to generalise on data from different CBCT systems [61];
- Employment of computational phantoms to make up for the lack of a ground truth by properly retraining the network [21,41];
- Exploration of the 3D variants of the networks, by either decreasing the FOV and reducing computational burden or by employing powerful cloud computing services that are compliant with data protection regulation [17];
- Inclusion of the other image similarity metrics by selecting specific regions of interest (ROIs) to bypass the problems related to possible anatomical mismatch [55];
- Dosimetric validation by means of dose difference pass rates (DPRs), gamma pass rates (GPRs) and dose–volume histogram (DVH)-based parameters [62,63];
- In vivo range verification and assessment of CT number accuracy of sCT to detect failures and outliers in the generated images [64];
- Evaluation of the capability of CBCT-based sCT to trigger replanning [4,15,56];
- Theoretical feasibility of integrating a sCT software in a clinical APT workflow [64,65], estimating the increase in clinical workload [66,67];
- Investigation of CycleGAN performances with different simulated CBCT imaging dose levels to assess the suitability of this method in low-dose paediatric protocols [68].

In recent years, AI applications in radiation oncology and medical physics have gained increasing popularity [11]. As stated by Spadea et al. [69], “*DL-based generation of sCT has a bright future, with extensive amount of research work being done on the topic*”. However, even if PT is more sensitive than photon radiotherapy to interfractional changes [70], the clinical integration of sCT into APT pathways remains circumscribed to few selected treatment sites and limited data [71]. A recent survey on the topic confirmed that the implementation of online daily APT is limited, with two centres only using the plan library [70]. A major obstacle is represented by the fact that CBCT-based APT pathways are not compatible with the fast clinical workflow and the small number of available clinical resources [72]. On the other hand, it was also experimentally demonstrated that APT requires a similar amount of time to that of a standard treatment delivery [73]. In general, a more structured cooperation between clinicians, researchers and industries is warranted to convert research outcomes into concrete clinical implementations.

## 5. Conclusions

In the era of personalised medicine and tailored treatments, APT is deemed to be a game changer. Despite the undisputable importance of adapting the treatment plan to the anatomy of the day, especially for high-variability districts and more sensitive populations (e.g., paediatrics), online adaptation approaches still lag behind and the interest of the scientific community is growing exponentially. In this scenario, this work represents a very preliminary attempt to improve the quality of the images obtained from non-commercial custom CBCT devices towards the generation of sCT scans for offline and online treatment adaptation in a real clinical environment. Despite the above-mentioned limitations to be addressed and future directions to be pursued, the present study adds to the current body of the literature highlighting the groundbreaking potential of DCNNs in generating a high-quality sCT even with narrow-FOV truncated CBCT as an input.

## Figures and Tables

**Table 1:** Summary of patients’ characteristics, including clinical, treatment and imaging data as well as training configuration information.

	Clinical Data					Treatment Data				Imaging Data		Training Configuration				
Study ID	Sex	Age at PT (Years)	Classification *	Treatment Site	Diagnosis	Particle	Nr. Fx	Dose Per Fx (GyRBE/fx)	Total Dose (GyRBE)	Nr. CTs	Nr CBCTs **	Nr. CBCTs Included	TR	TS	Discarded	Reason for Discarding
**PT_01**	F	15	Paediatric	Sacrum	Ewing’s Sarcoma	P	33	1.8 × 28 + 1.8 × 5 (boost)	59.4	7	6	6	6			
**PT_02**	M	16	Paediatric	Right Sacrum	Osteosarcoma	C	16	4.6 × 9 + 4.6 × 7 (boost)	73.6	3	2	0			2	CT import
**PT_03**	M	10	Paediatric	Right Iliac Crest	Ewing’s Sarcoma	P	33	1.8 × 28 + 1.8 × 5 (boost)	59.4	5	4	4	4			
**PT_04**	M	15	Paediatric	Left Pelvis	Osteosarcoma (relapse)	C	16	4.2 × 16	67.2	5	3	3	3			
**PT_05**	M	15	Paediatric	Left Pelvis	Osteosarcoma (relapse)	C	15	4 × 15	60	3	2	2		2		
**PT_06**	M	15	Paediatric	Sacrum-Coccyx	Ewing’s Sarcoma	P	33	1.8 × 30 + 1.8 × 3 (boost)	59.4	7	6	5	5		1	Registration
**PT_07**	M	14	Paediatric	Sacrum-Coccyx	Ewing’s Sarcoma	P	33	1.8 × 30 + 1.8 × 3 (boost)	59.4	7	6	6	6			
**PT_08**	F	16	Paediatric	Left Gluteus	Rhabdomyosarcoma	P	31	1.8 × 28 + 1.8 × 3 (boost)	55.8	4	3	3		3		
**PT_09**	M	7	Paediatric	Prostate	Rhabdomyosarcoma	P	30	1.8 × 30	54	3	3	0			3	Image quality
**PT_10**	F	16	Paediatric	Right Gluteus	Sarcoma	P	28	1.8 × 28	50.4	4	3	3	3			
**PT_11**	F	17	Paediatric	Sacrum	Ewing’s sarcoma (relapse)	C	15	4 × 15	60	2	1	1	1			
**PT_12**	M	17	Paediatric	Right Pelvis	Ewing’s Sarcoma	P	33	1.8 × 33	59.4	6	5	5	5			
**PT_13**	F	20	Young Adult	Sacrum/Left Iliac Crest	Osteosarcoma	C	16	4.6 × 16	73.6	2	1	1		1		
**PT_14**	F	20	Young Adult	Right Sciatic Nerve	MPNST	C	16	4.6 × 16	73.6	2	1	0			1	CT import
**PT_15**	M	24	Young Adult	Sacrum-Coccyx	Cordoma	C	16	4.8 × 13 + 4.8 × 3 (boost)	76.8	2	1	1		1		
**PT_16**	F	27	Young Adult	Right Iliac Crest	Condrosarcoma	C	16	4.6 × 16	73.6	2	1	1	1			
**PT_17**	M	27	Young Adult	Pelvis	Sarcoma	C	15	-	-	3	0	0				
**PT_18**	F	27	Young Adult	Left Pelvis	Sarcoma	C	12	4.6 × 12	55.2	2	1	0			1	Registration
**PT_19**	F	28	Young Adult	Vagina	Adenocarcinoma	C	8	4.8 × 8	38.4	3	2	2	2			
**PT_20**	F	30	Young Adult	Sacrum	Emangioma	P	30	2 × 30	60	4	3	1		1	2	
**PT_21**	M	30	Young Adult	Pelvis	Osteosarcoma	C	16	4.8 × 13	62.4	3	1	0			1	Registration
									**TOT**	**79**	**55**	**44**	**36**	**8**	**11**	

* Age ≤ 18: paediatric; 18 ≤ Age ≤ 30: young adult. ** Same-day CBCT scans (one-day distance, at most). **Abbreviations:** C: carbon ion; CBCT: cone beam computed tomography; CT: computed tomography; F: female; Fx: fraction; Gy: grey; ID: identification number; M: male; Nr: number; P: proton; PT: particle therapy; PT_: patient; RBE: relative biological effectiveness; TR: training; TS: testing; X: discarded.

**Table 2:** Image quality quantitative evaluation.

			Res-Net		U-Net	
			SSIM (Median, IQR)	PSNR (Median, IQR)	SSIM (Median, IQR)	PSNR (Median, IQR)
**PT_05**	**V1**	**Pre**	0.73, 0.03	49.09, 2.88	0.73, 0.03	49.09, 2.88
		**Post**	0.79, 0.05	51.97, 2.96	0.75, 0.04	51.39, 3.67
		***p*-value**	<0.05	<0.05	<0.05	<0.05
	**V2**	**Pre**	0.69, 0.05	48.48, 2.70	0.69, 0.05	48.48, 2.70
		**Post**	0.73, 0.07	50.68, 3.85	0.72, 0.06	50.27, 3.94
		***p*-value**	<0.05	<0.05	<0.05	<0.05
**PT_08**	**V1**	**Pre**	0.58, 0.17	49.85, 1.93	0.58, 0.17	49.85, 1.93
		**Post**	0.57, 0.20	50.90, 1.60	0.57, 0.21	50.28, 2.25
		***p*-value**	<0.05	<0.05	0.19	<0.05
	**V2**	**Pre**	0.54, 0.18	49.69, 1.82	0.54, 0.18	49.69, 1.82
		**Post**	0.54, 0.19	50.42, 0.72	0.54, 0.21	50.32, 1.33
		***p*-value**	<0.05	<0.05	0.41	<0.05
	**V3**	**Pre**	0.55, 0.17	49.02, 1.31	0.55, 0.17	49.02, 1.31
		**Post**	0.54, 0.20	49.46, 0.21	0.54, 0.20	49.48, 1.00
		***p*-value**	<0.05	<0.05	0.90	<0.05
**PT_13**		**Pre**	0.75, 0.04	48.66, 2.19	0.75, 0.04	48.66, 2.19
		**Post**	0.83, 0.02	52.96, 3.47	0.78, 0.04	50.73, 2.34
		***p*-value**	<0.05	<0.05	<0.05	<0.05
**PT_15**		**Pre**	0.75, 0.03	48.24, 2.14	0.75, 0.03	48.24, 2.14
		**Post**	0.80, 0.03	52.95, 3.02	0.78, 0.05	50.48, 1.60
		***p*-value**	<0.05	<0.05	<0.05	<0.05
**PT_20**		**Pre**	0.68, 0.07	44.40, 2.10	0.68, 0.07	44.40, 2.10
		**Post**	0.73, 0.08	48.98, 2.89	0.73, 0.06	48.17, 2.05
		***p*-value**	<0.05	<0.05	<0.05	<0.05

**Legend:** pre = rCBCT-vCT; post = sCT-vCT. **Abbreviations:** IQR: interquartile range; PT_: patient; PSNR: peak signal-to-noise ratio; SSIM: structural similarity; V: volume.

## Data Availability

Restrictions apply to the dataset.

## Supplementary Materials

The following supporting information can be downloaded at: https://www.mdpi.com/article/10.3390/s24237460/s1, Figure S1: Dataset. Figure S2: Slice-wise SSIM values before (rCBCT vs vCT) and after (sCT vs vCT) CycleGAN for both Res-Net (left) and U-Net (right) architectures. Median values are also reported. Figure S3: SSIM (up) and PSNR (down) values distribution before (vCT vs rCBCT) and after (vCT vs sCT) CycleGAN, for both architectures (Res-Net, left and U-Net, right). Median values are also reported.

## List of Abbreviations

**AI**	*Artificial Intelligence*
**APT**	*Adaptive PT*
**c**	*Contrast*
**C**	*Carbon ion*
**CBCT**	*Cone beam CT*
**CNAO**	*Centro Nazionale di Adroterapia Oncologica (National Centre for Oncological Hadrontherapy)*
**CT**	*Computed tomography*
**D_CBCT_**	*CBCT discriminator*
**D_CT_**	*CT discriminator*
**DCNN**	*Deep convolutional neural network*
**DFT**	*Discrete Fourier transform*
**DIR**	*Deformable image registration*
**DL**	*Deep learning*
**DPR**	*Dose Pass Rate*
**DVH**	*Dose–volume histogram*
**E**	*Expected value*
**F**	*Female*
**fCBCT**	*Filtered CBCT*
**FDK**	*Feldkamp–Davis–Kress (algorithm)*
**FF**	*Full-fan (CBCT)*
**FOV**	*Field of view*
**Fx**	*Fraction*
**GAN**	*Generative adversarial network*
**GPR**	*Gamma pass rate*
**GPU**	*Graphical processing unit*
**Gy**	*Grey*
**HF**	*Half-fan (CBCT)*
**HU**	*Hounsfield unit*
**iCBCT**	*Input CBCT*
**l**	*Luminance*
**L**	*Full objective*
**L_IDENTITY_**	*Identity loss*
**L_GAN-F_**	*Adversarial loss (F: CBCT generator)*
**L_GAN-G_**	*Adversarial loss (G: CT generator)*
**M**	*Male*
**MAE**	*Mean absolute error*
**MRI**	*Magnetic resonance imaging*
**MSE**	*Mean square error*
**OAR**	*Organ at risk*
**P**	*Proton*
**pCT**	*Planning CT*
**PSNR**	*Peak signal-to-noise ratio*
**PT**	*Particle therapy*
**PT_**	*Patient*
**RBE**	*Relative Biological Effectiveness*
**rCBCT**	*CBCT registered (on CT)*
**RMSE**	*Root mean square error*
**RR**	*Rigid registration*
**s**	*Structure*
**SSIM**	*Structural similarity*
**sCT**	*Synthetic CT*
**TPS**	*Treatment Planning System*
**TR**	*Training*
**TS**	*Testing*
**vCT**	*Verification CT*
**X**	*Discarded*
**Yo**	*Years old*
