# Supplementary material for: Unsupervised Deep Learning for Synthetic CT Generation from CBCT Images for Proton and Carbon Ion Therapy for Paediatric Patients
Source: Sensors (Basel). 2024 Nov 22;24(23):7460. doi: 10.3390/s24237460 (PMC11644336; doi:10.3390/s24237460)
Supplement: Supplementary file 1 [file sensors-24-07460-s001.zip › sensors-3249178-supplementary.pdf]

sensors

MDPI

Supplementary material

Figure S1. Dataset

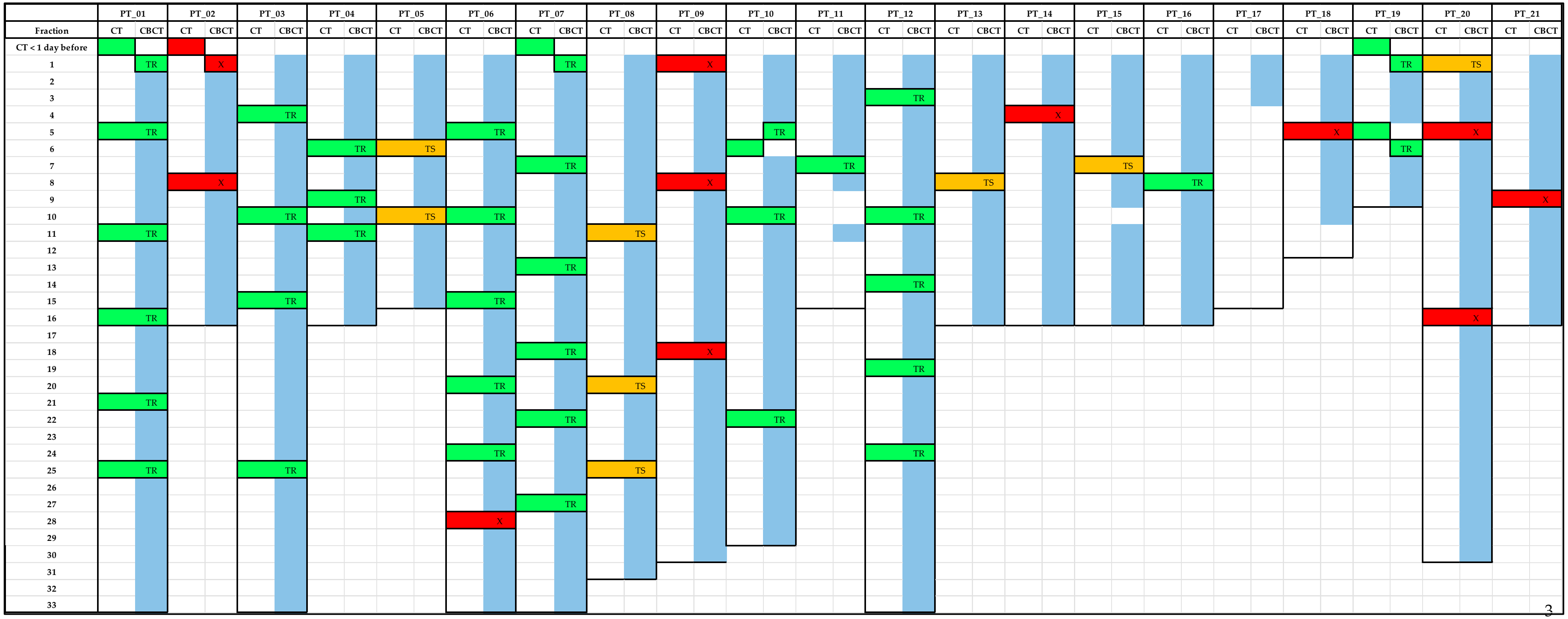

Legend: Light blue: all available CBCTs; green: same-day CBCTs used for training (TR); orange: same-day CBCTs used for testing (TS); red: same day CBCTs discarded (X).

Abbreviations: CBCT: cone beam computed tomography; CT: computed tomography; PT_: patient.

sensors

MDPI

Figure S2. Slice-wise SSIM values before (rCBCT vs vCT) and after (sCT vs vCT) cycleGAN for both Res-Net (left) and U-Net (right) architectures. Median values are also reported.

Res-Net **U-Net**

PT_05 V1

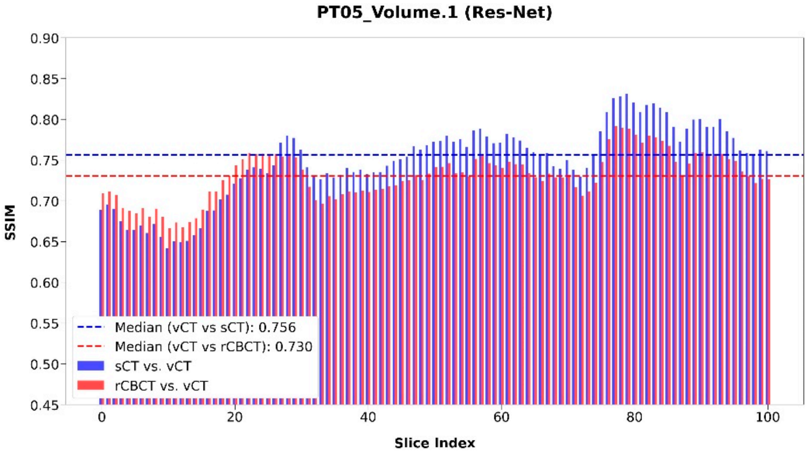

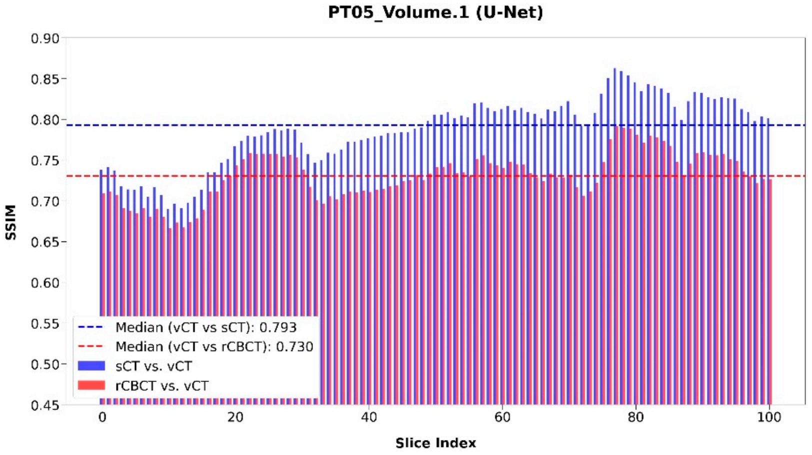

V2

PT_08 V1

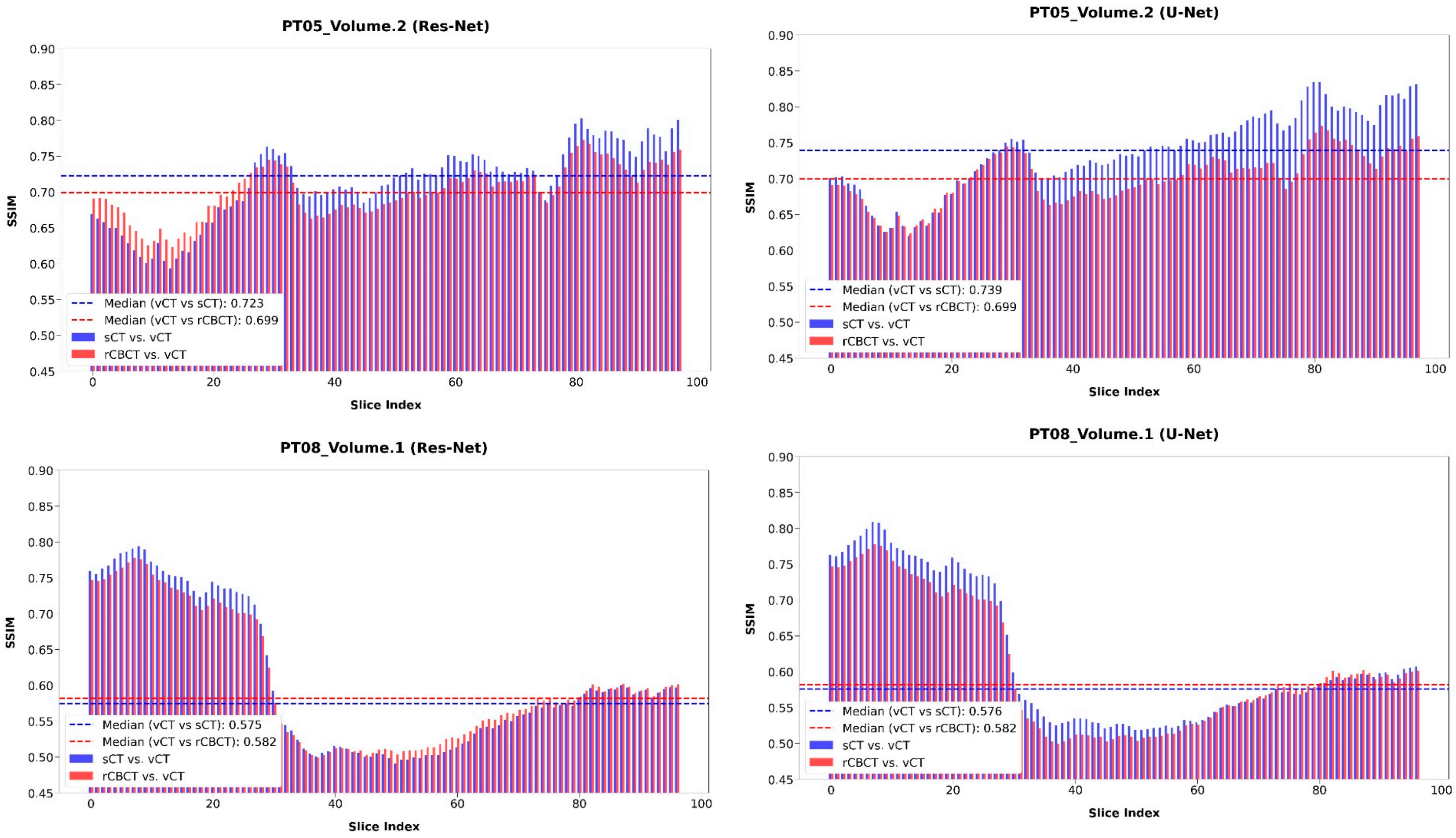
PT05_Volume.2 (Res-Net)
Median (vCT vs sCT): 0.723
Median (vCT vs rCBCT): 0.699
sCT vs. vCT
rCBCT vs. vCT
SSIM
Slice Index
PT05_Volume.2 (U-Net)
Median (vCT vs sCT): 0.739
Median (vCT vs rCBCT): 0.699
sCT vs. vCT
rCBCT vs. vCT
SSIM
Slice Index
PT08_Volume.1 (Res-Net)
Median (vCT vs sCT): 0.575
Median (vCT vs rCBCT): 0.582
sCT vs. vCT
rCBCT vs. vCT
SSIM
Slice Index
PT08_Volume.1 (U-Net)
Median (vCT vs sCT): 0.576
Median (vCT vs rCBCT): 0.582
sCT vs. vCT
rCBCT vs. vCT
SSIM
Slice Index

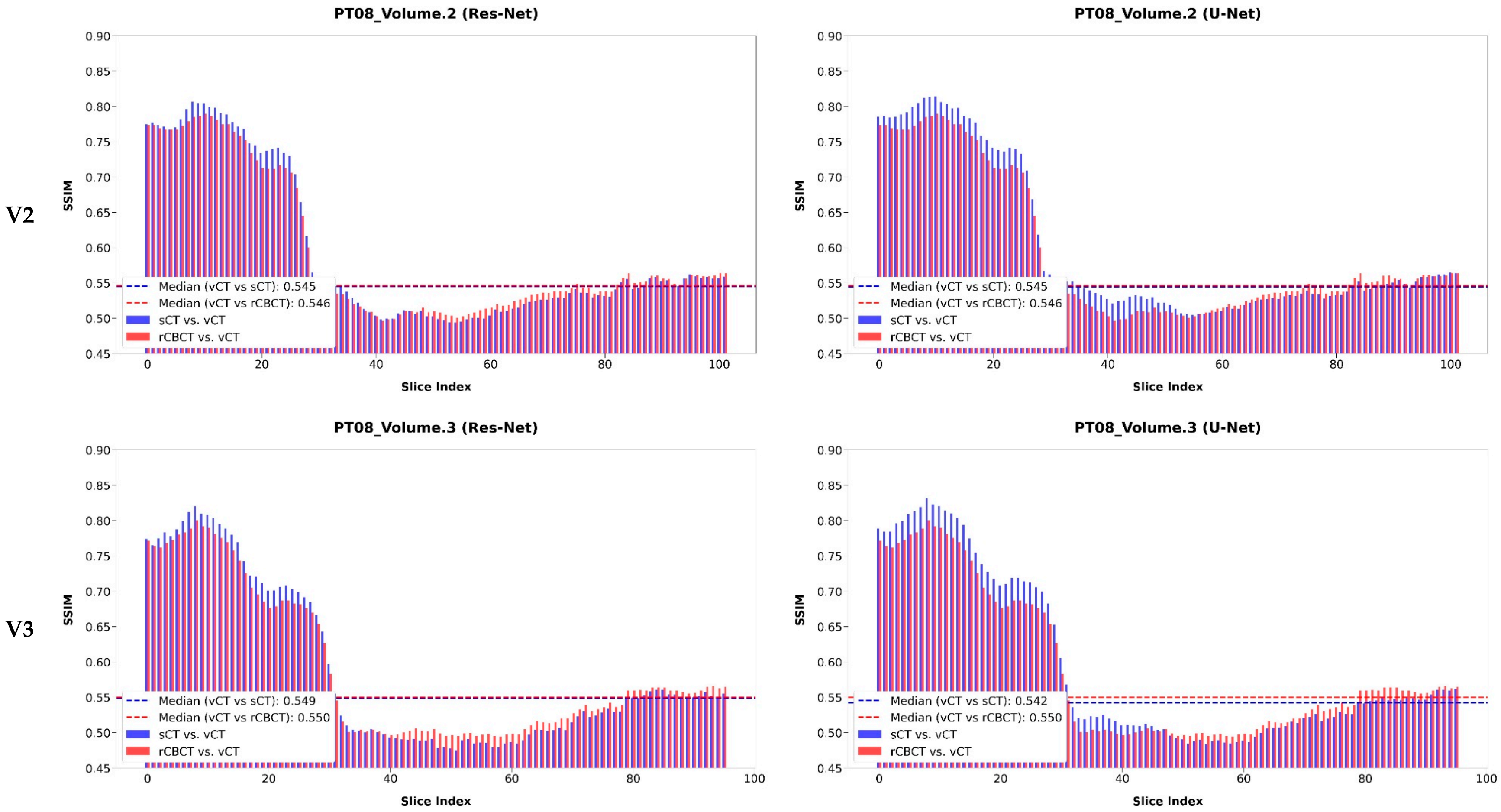
V2
V3
PT08_Volume.2 (Res-Net)
PT08_Volume.2 (U-Net)
PT08_Volume.3 (Res-Net)
PT08_Volume.3 (U-Net)
SSIM
Slice Index
Median (vCT vs sCT): 0.545
Median (vCT vs rCBCT): 0.546
Median (vCT vs sCT): 0.549
Median (vCT vs rCBCT): 0.550
Median (vCT vs sCT): 0.542
sCT vs. vCT
rCBCT vs. vCT

PT_13

PT_15

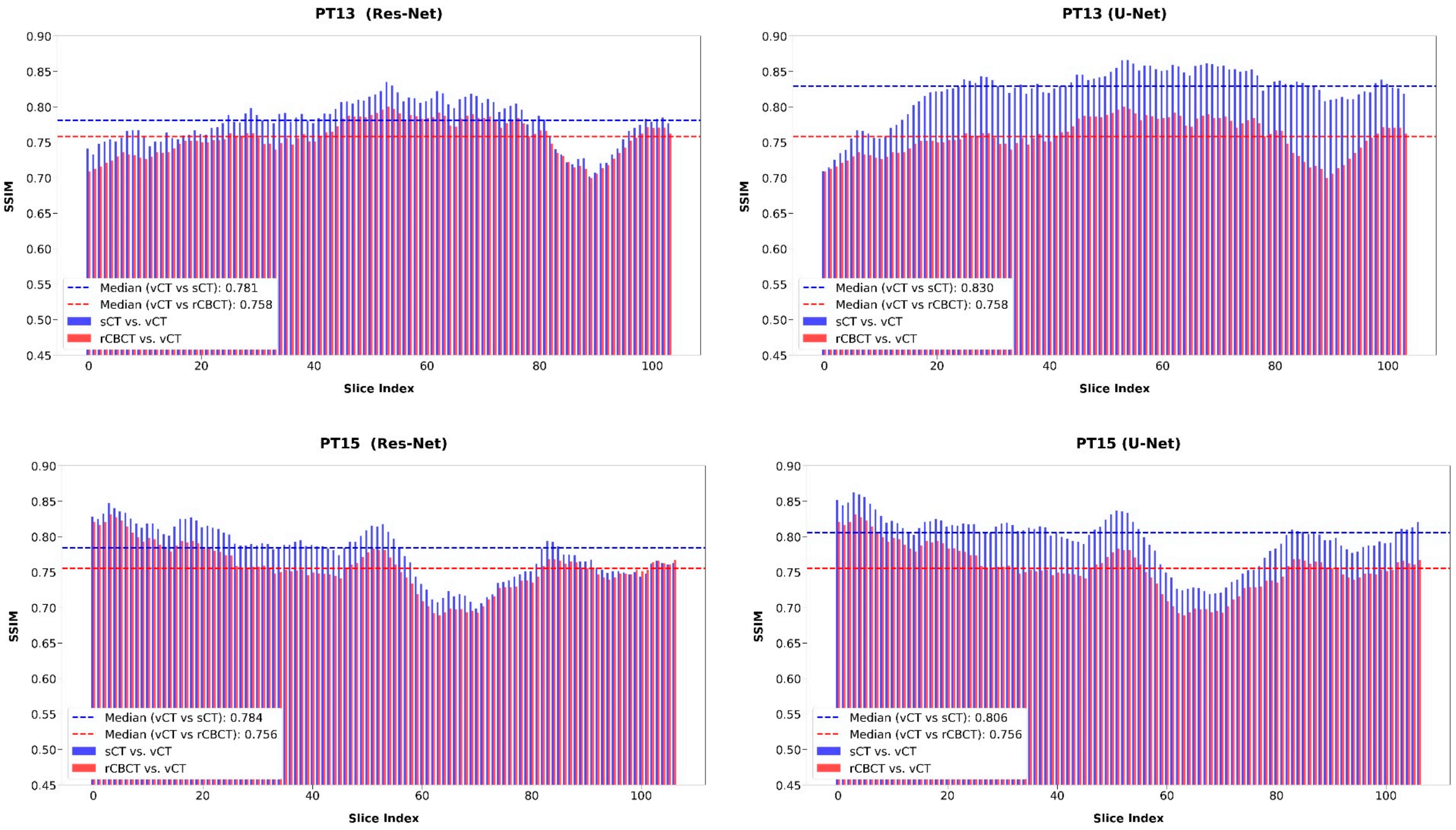
PT13 (Res-Net)
Median (vCT vs sCT): 0.781
Median (vCT vs rCBCT): 0.758
sCT vs. vCT
rCBCT vs. vCT
SSIM
Slice Index
PT13 (U-Net)
Median (vCT vs sCT): 0.830
Median (vCT vs rCBCT): 0.758
sCT vs. vCT
rCBCT vs. vCT
SSIM
Slice Index
PT15 (Res-Net)
Median (vCT vs sCT): 0.784
Median (vCT vs rCBCT): 0.756
sCT vs. vCT
rCBCT vs. vCT
SSIM
Slice Index
PT15 (U-Net)
Median (vCT vs sCT): 0.806
Median (vCT vs rCBCT): 0.756
sCT vs. vCT
rCBCT vs. vCT
SSIM
Slice Index

PT_20

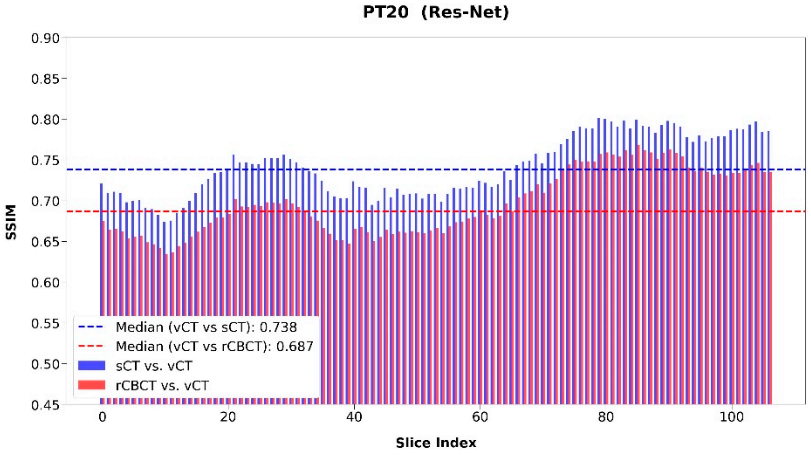
PT20 (Res-Net)
SSIM
Slice Index
Median (vCT vs sCT): 0.738
Median (vCT vs rCBCT): 0.687
sCT vs. vCT
rCBCT vs. vCT

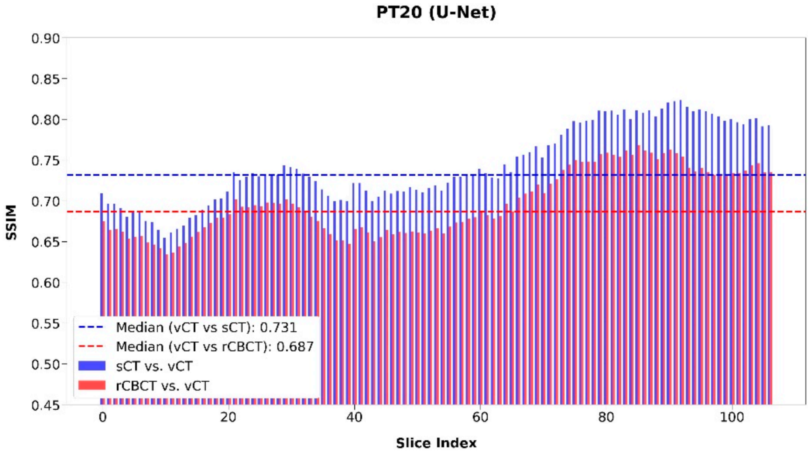
PT20 (U-Net)
SSIM
Slice Index
Median (vCT vs sCT): 0.731
Median (vCT vs rCBCT): 0.687
sCT vs. vCT
rCBCT vs. vCT

9

Figure S3. SSIM (up) and PSNR (down) values distribution before (vCT vs rCBCT) and after (vCT vs sCT) cycleGAN, for both architectures (Res-Net, left and U-Net, right). Median values are also reported.

			Res-Net	U-Net
PT_05	V1	SSIM	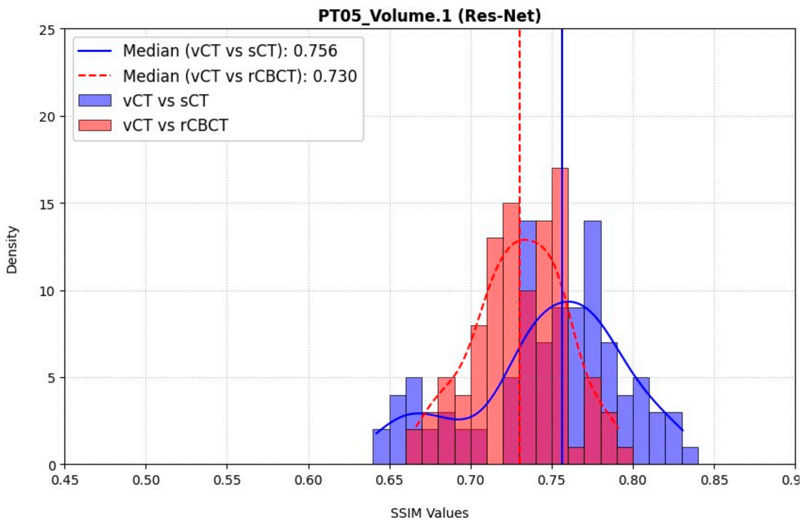	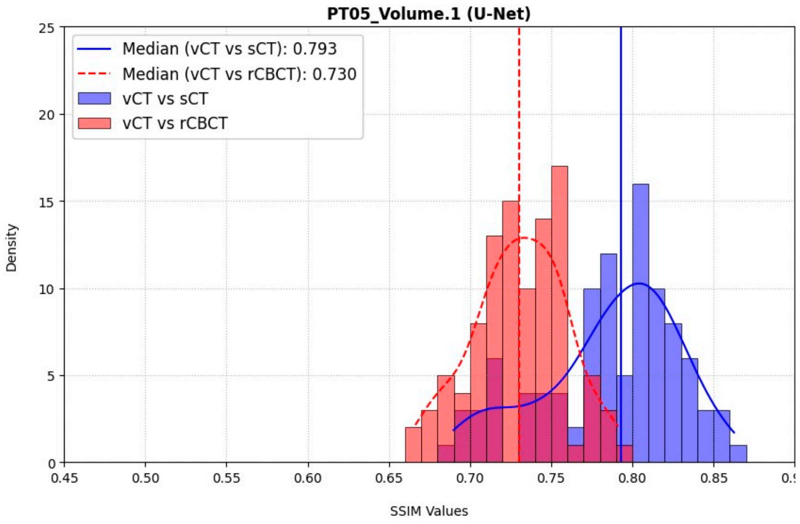

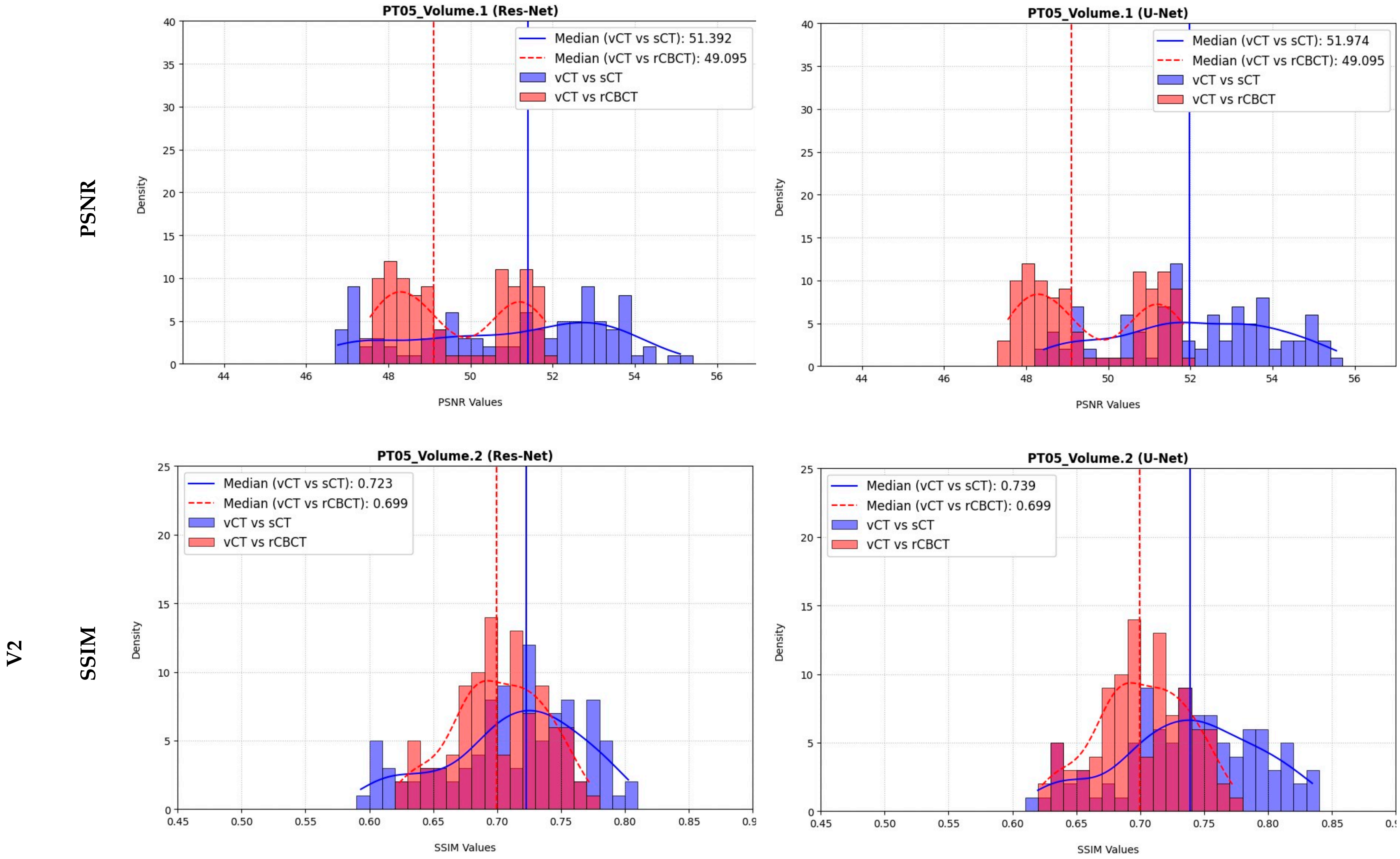
PSNR
SSIM
V2
PT05_Volume.1 (Res-Net)
Median (vCT vs sCT): 51.392
Median (vCT vs rCBCT): 49.095
vCT vs sCT
vCT vs rCBCT
PSNR Values
Density
PT05_Volume.1 (U-Net)
Median (vCT vs sCT): 51.974
Median (vCT vs rCBCT): 49.095
vCT vs sCT
vCT vs rCBCT
PSNR Values
Density
PT05_Volume.2 (Res-Net)
Median (vCT vs sCT): 0.723
Median (vCT vs rCBCT): 0.699
vCT vs sCT
vCT vs rCBCT
SSIM Values
Density
PT05_Volume.2 (U-Net)
Median (vCT vs sCT): 0.739
Median (vCT vs rCBCT): 0.699
vCT vs sCT
vCT vs rCBCT
SSIM Values
Density

PSNR

PT_08 V1 SSIM

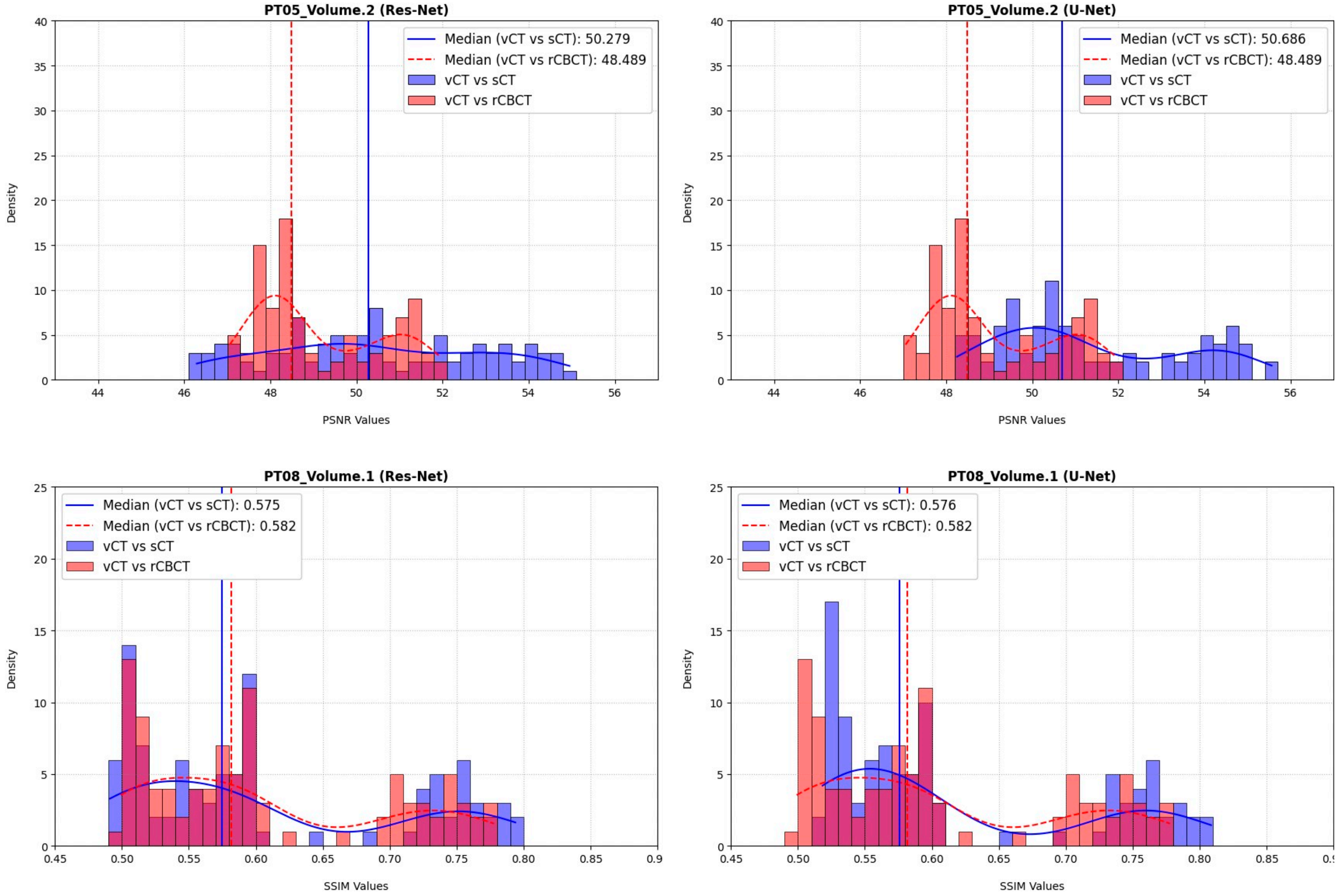

PSNR

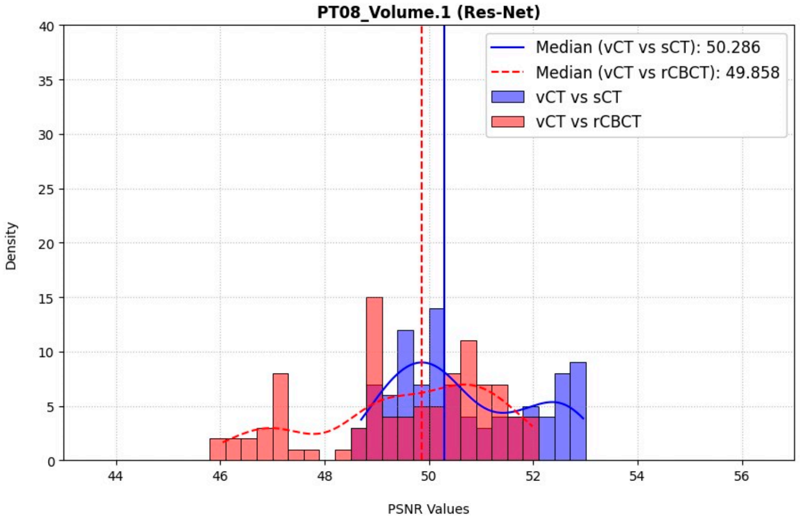
PT08_Volume.1 (Res-Net)
Median (vCT vs sCT): 50.286
Median (vCT vs rCBCT): 49.858
vCT vs sCT
vCT vs rCBCT
Density
PSNR Values

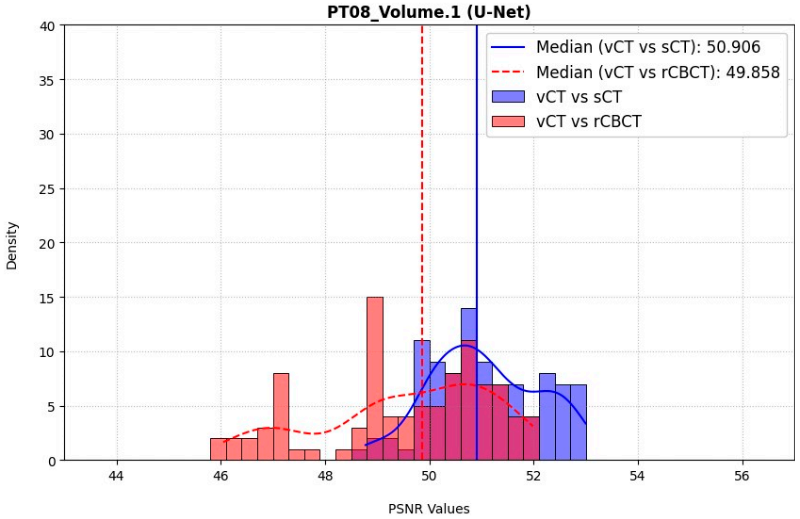
PT08_Volume.1 (U-Net)
Median (vCT vs sCT): 50.906
Median (vCT vs rCBCT): 49.858
vCT vs sCT
vCT vs rCBCT
Density
PSNR Values

V2

SSIM

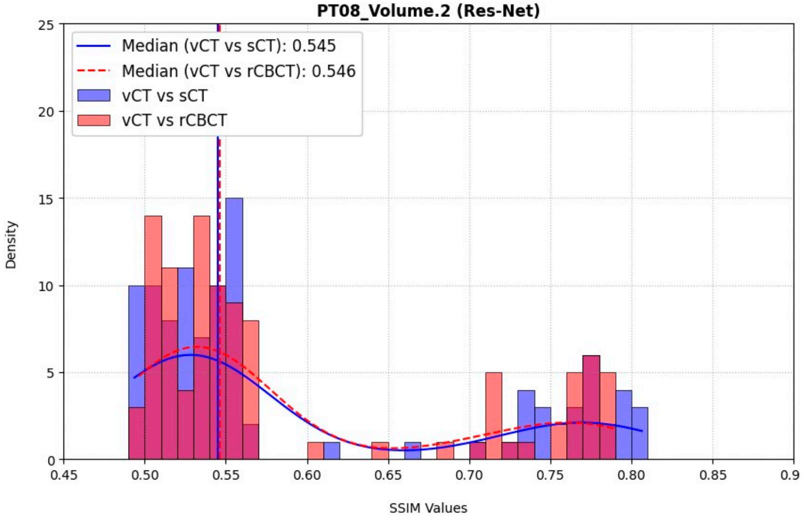
PT08_Volume.2 (Res-Net)
Median (vCT vs sCT): 0.545
Median (vCT vs rCBCT): 0.546
vCT vs sCT
vCT vs rCBCT
Density
SSIM Values

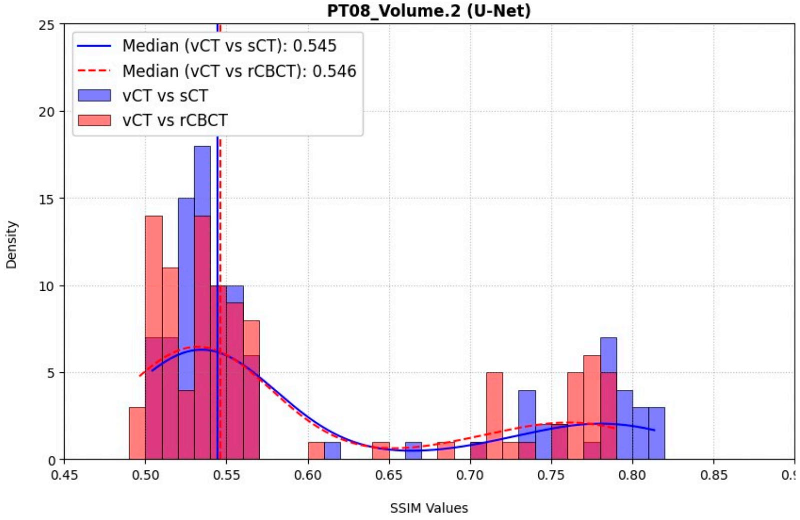
PT08_Volume.2 (U-Net)
Median (vCT vs sCT): 0.545
Median (vCT vs rCBCT): 0.546
vCT vs sCT
vCT vs rCBCT
Density
SSIM Values

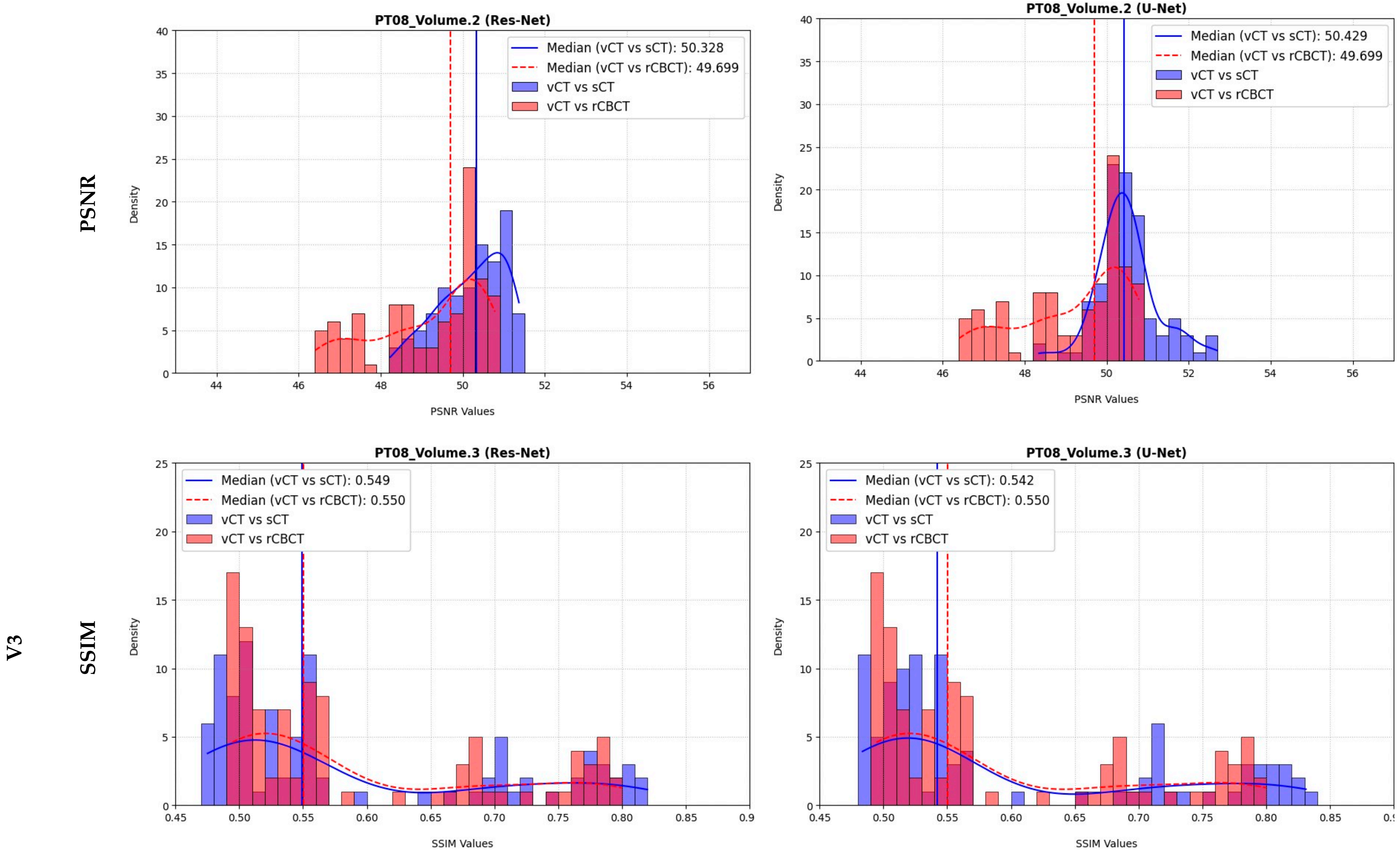
PSNR
V3
SSIM
PT08_Volume.2 (Res-Net)
Median (vCT vs sCT): 50.328
Median (vCT vs rCBCT): 49.699
vCT vs sCT
vCT vs rCBCT
Density
PSNR Values
PT08_Volume.2 (U-Net)
Median (vCT vs sCT): 50.429
Median (vCT vs rCBCT): 49.699
vCT vs sCT
vCT vs rCBCT
Density
PSNR Values
PT08_Volume.3 (Res-Net)
Median (vCT vs sCT): 0.549
Median (vCT vs rCBCT): 0.550
vCT vs sCT
vCT vs rCBCT
Density
SSIM Values
PT08_Volume.3 (U-Net)
Median (vCT vs sCT): 0.542
Median (vCT vs rCBCT): 0.550
vCT vs sCT
vCT vs rCBCT
Density
SSIM Values

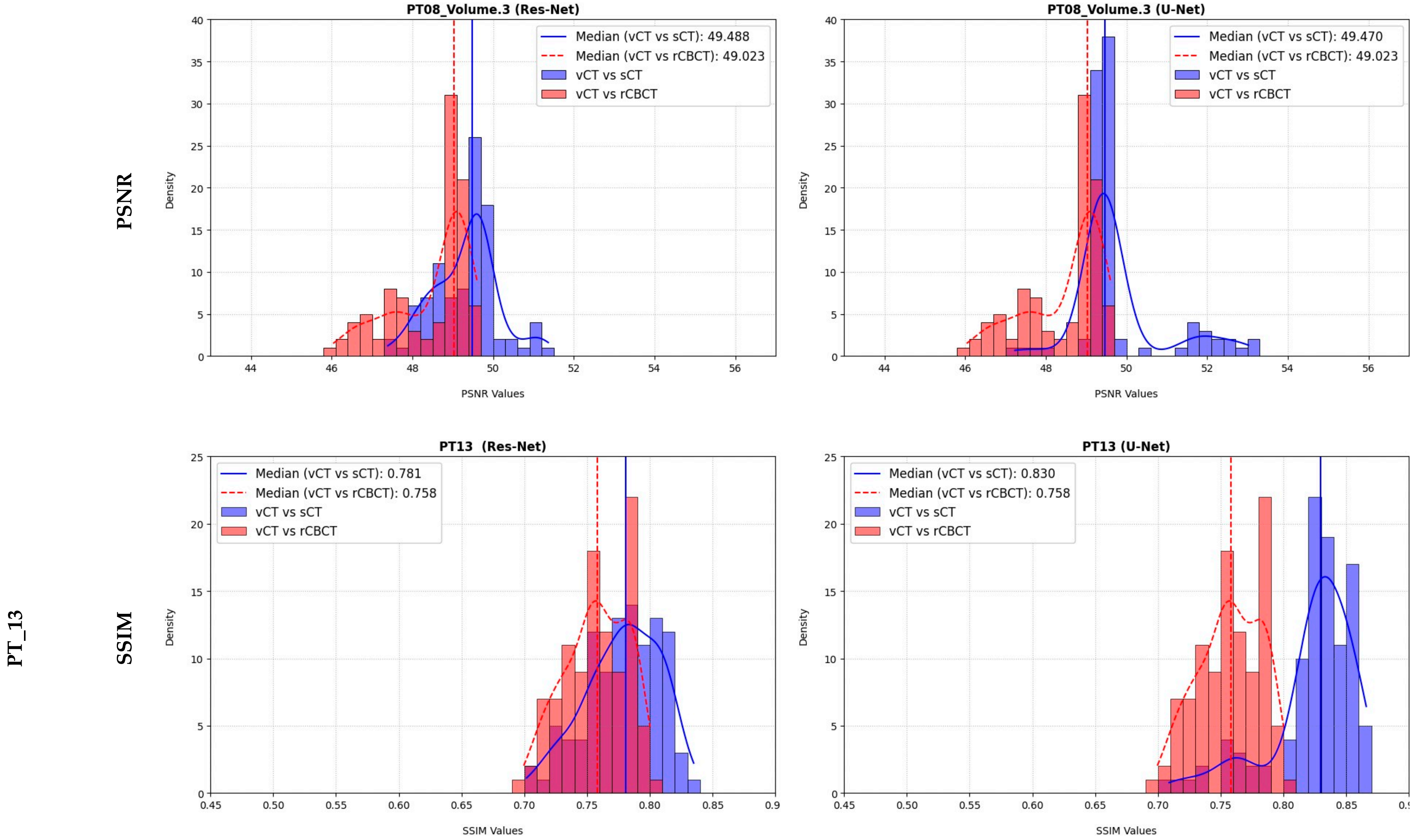
PSNR
SSIM
PT_13
PT08_Volume.3 (Res-Net)
Median (vCT vs sCT): 49.488
Median (vCT vs rCBCT): 49.023
vCT vs sCT
vCT vs rCBCT
Density
PSNR Values
PT08_Volume.3 (U-Net)
Median (vCT vs sCT): 49.470
Median (vCT vs rCBCT): 49.023
vCT vs sCT
vCT vs rCBCT
Density
PSNR Values
PT13 (Res-Net)
Median (vCT vs sCT): 0.781
Median (vCT vs rCBCT): 0.758
vCT vs sCT
vCT vs rCBCT
Density
SSIM Values
PT13 (U-Net)
Median (vCT vs sCT): 0.830
Median (vCT vs rCBCT): 0.758
vCT vs sCT
vCT vs rCBCT
Density
SSIM Values

PT_15

PSNR

SSIM

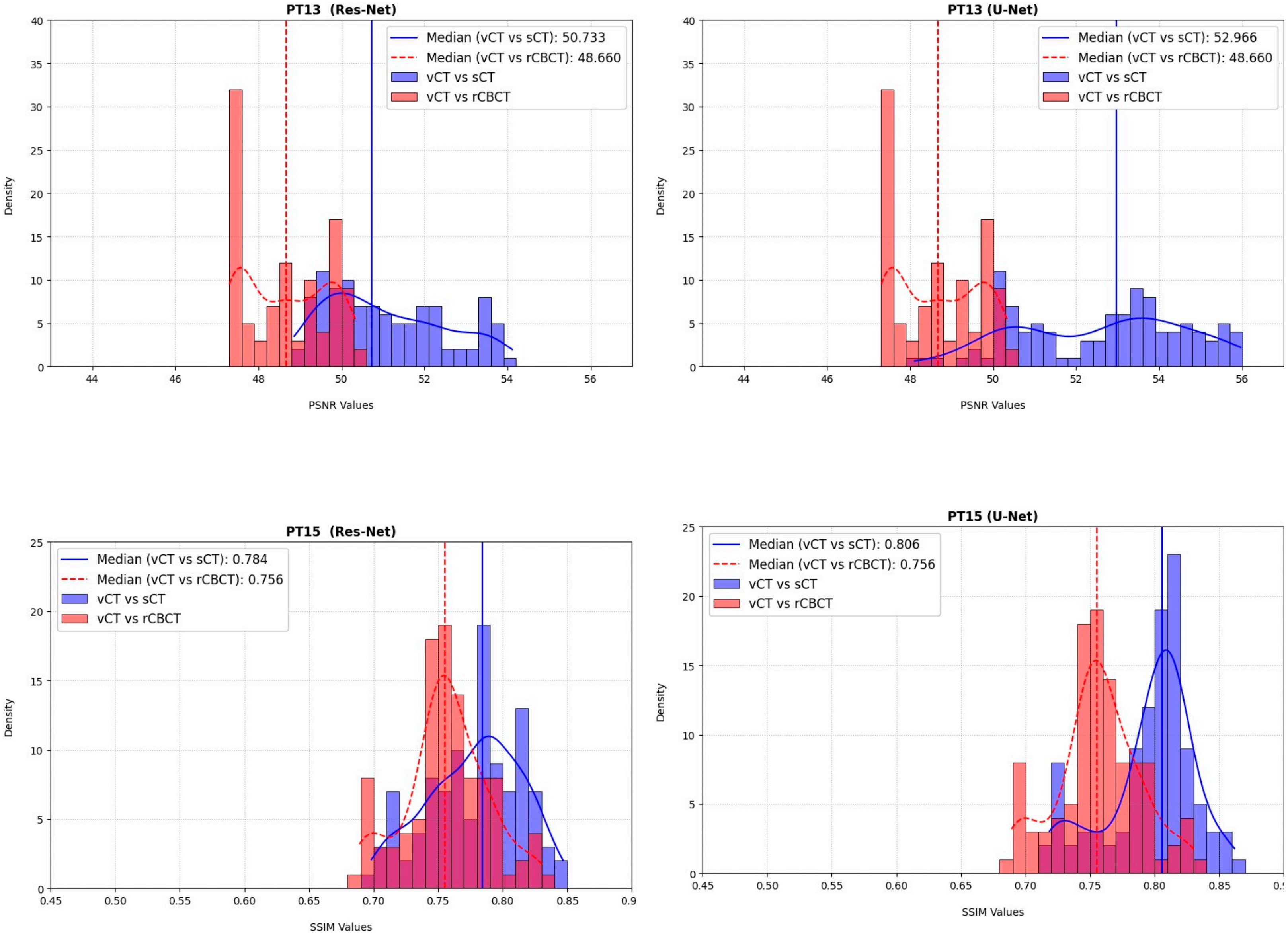

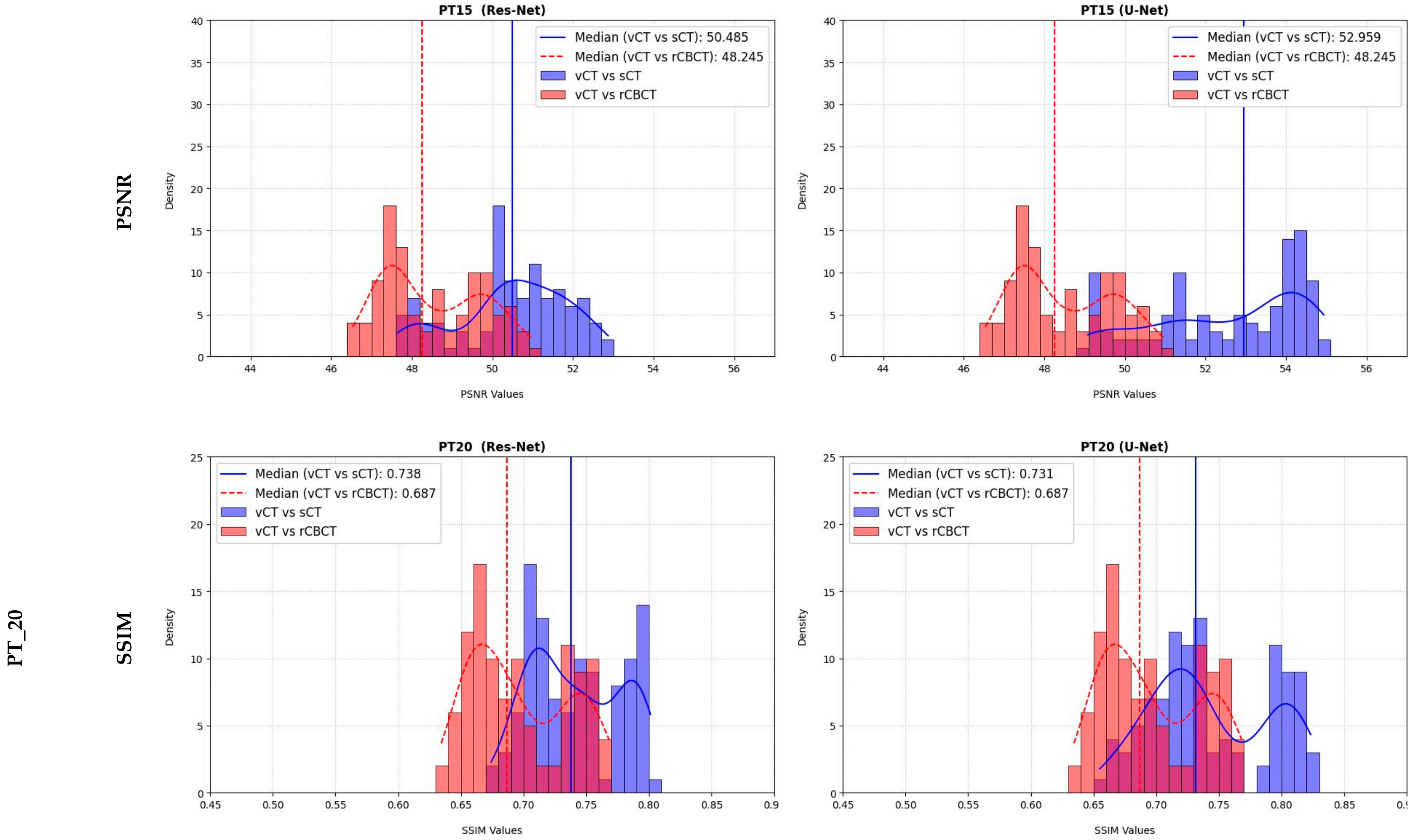
PT_20
PSNR
SSIM
PT15 (Res-Net)
Median (vCT vs sCT): 50.485
Median (vCT vs rCBCT): 48.245
vCT vs sCT
vCT vs rCBCT
Density
PSNR Values
PT15 (U-Net)
Median (vCT vs sCT): 52.959
Median (vCT vs rCBCT): 48.245
vCT vs sCT
vCT vs rCBCT
Density
PSNR Values
PT20 (Res-Net)
Median (vCT vs sCT): 0.738
Median (vCT vs rCBCT): 0.687
vCT vs sCT
vCT vs rCBCT
Density
SSIM Values
PT20 (U-Net)
Median (vCT vs sCT): 0.731
Median (vCT vs rCBCT): 0.687
vCT vs sCT
vCT vs rCBCT
Density
SSIM Values

PSNR

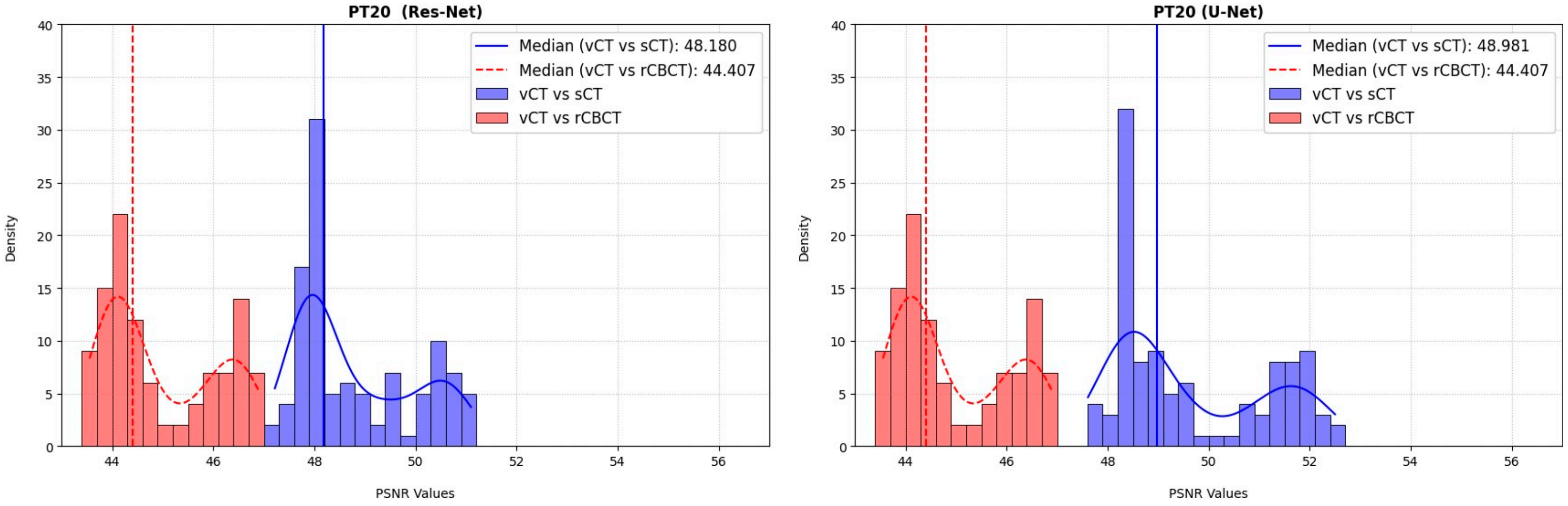
PT20 (Res-Net)
Median (vCT vs sCT): 48.180
Median (vCT vs rCBCT): 44.407
vCT vs sCT
vCT vs rCBCT
Density
PSNR Values
PT20 (U-Net)
Median (vCT vs sCT): 48.981
Median (vCT vs rCBCT): 44.407
vCT vs sCT
vCT vs rCBCT
Density
PSNR Values
